# Principles of glycocalyx engineering with hydrophobic-anchored synthetic mucins

**DOI:** 10.3389/fcell.2022.952931

**Published:** 2022-10-17

**Authors:** Casia L. Wardzala, Zachary S. Clauss, Jessica R. Kramer

**Affiliations:** Department of Biomedical Engineering, University of Utah, Salt Lake City, UT, United States

**Keywords:** mucin, glycocalyx, cell-surface engineering, glycopolymer, glycopolypeptide

## Abstract

The cellular glycocalyx is involved in diverse biological phenomena in health and disease. Yet, molecular level studies have been challenged by a lack of tools to precisely manipulate this heterogeneous structure. Engineering of the cell surface using insertion of hydrophobic-terminal materials has emerged as a simple and efficient method with great promise for glycocalyx studies. However, there is a dearth of information about how the structure of the material affects membrane insertion efficiency and resulting density, the residence time of the material, or what types of cells can be utilized. Here, we examine a panel of synthetic mucin structures terminated in highly efficient cholesterylamide membrane anchors for their ability to engineer the glycocalyx of five different cell lines. We examined surface density, residence time and half-life, cytotoxicity, and the ability be passed to daughter cells. We report that this method is robust for a variety of polymeric structures, long-lasting, and well-tolerated by a variety of cell lines.

## Introduction

All cells are covered with a diverse array of carbohydrates, termed glycans, attached to proteins and lipids. These sugar-coated structures collectively form the cellular glycocalyx. This structure is involved in nearly all aspects of biology. The glycocalyx has bulk physical properties as a lubricating barrier structure, however, the glycans are also biochemically active in diverse physiological processes. Notable examples are embryonic and neural cell development (Lanctot, Gage and Varki, 2007; Henderson-Toth et al., 2012; Huang and Godula, 2014), cancer progression (Rambaruth and Dwek, 2011), immune cell activation (Rabinovich and Toscano, 2009; Tsuboi et al., 2012; Möckl, 2020), and pathogen attenuation (Wheeler et al., 2019; Wardzala et al., 2022).

Mucin family glycoproteins are the major component of the epithelial glycocalyx and in bulk, form mucus (Meldrum et al., 2018). More than 20 mucin genes have been identified, and even more proteins have mucin-like domains. The key characteristics of mucins are high molecular weight (MW), heavily glycosylated domains. These glycodomains are rigid and extended in a polyproline (PP) II helical conformation due to a high proline and glycosylated serine and threonine content (Kramer et al., 2015). Mucin glycosylation initiates with α*-N*-acetylgalactosamine (αGalNAc) and additional sugars are appended on its 3-, 4-, and/or 6-hydroxyls. Glycan patterns are the result of complex metabolic pathways involving over a thousand genes (Hattrup and Gendler, 2008; Guzman-Aranguez et al., 2012). Therefore, mucin glycosylation is dynamic and varied where proteins originating from the same gene can have different structures due to varied glycosylation as well as alternative splicing. Due to this complexity, and a lack of biochemical and genetic manipulation methods, mucins and other glycocalyx constituents have been difficult to define in terms of their molecular structure-function relationships. Consequently, researchers have investigated a variety of synthetic mucin-mimetic structures along with methods to display them on cell-surfaces. These approached have shed light on processes from oncogenesis (Paszek et al., 2014; Woods et al., 2017) and neural development (Huang et al., 2014; Pulsipher et al., 2014) to infection processes (Delaveris et al., 2020; Honigfort et al., 2021).

**FIGURE 1:**
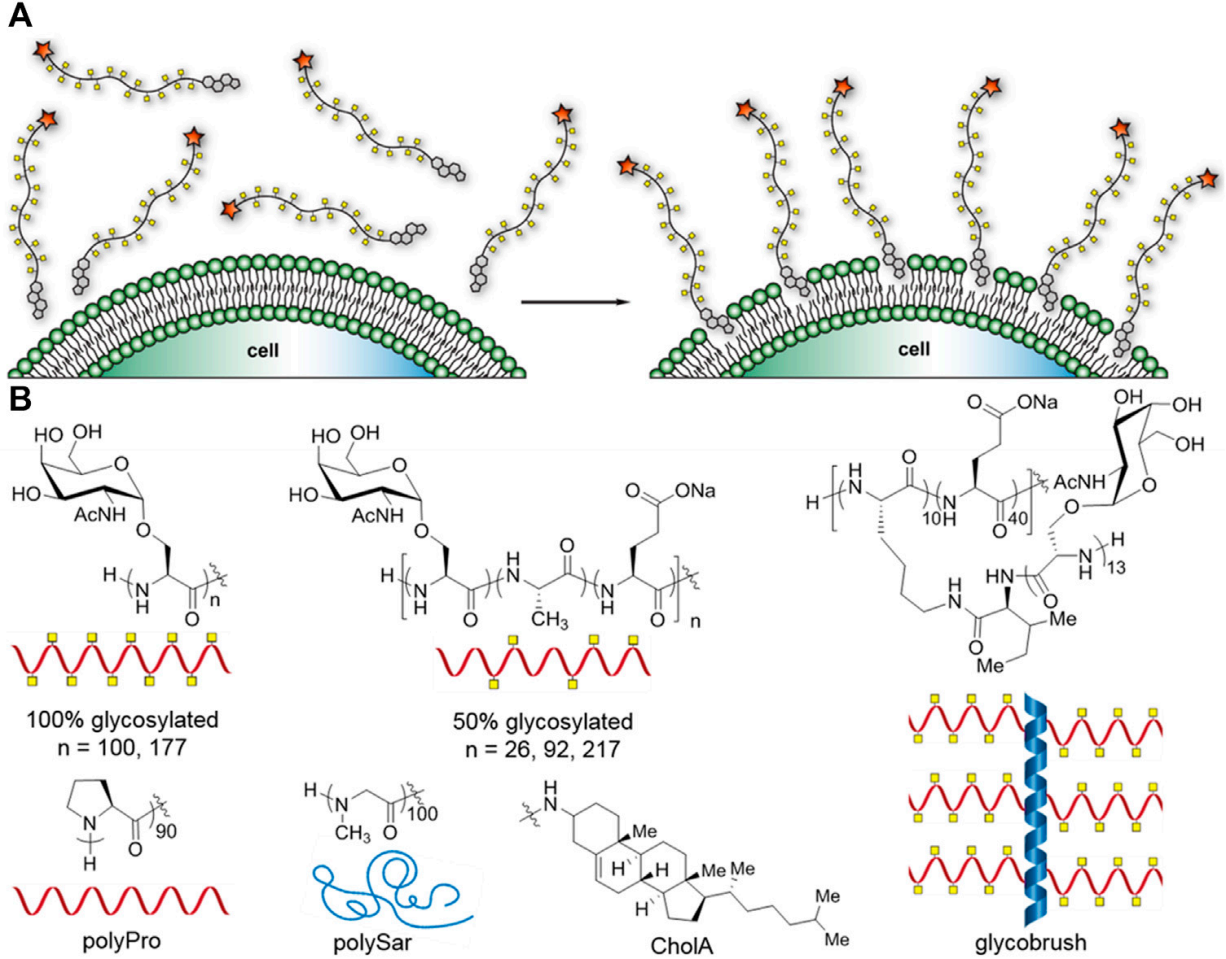
Cartoon representation of the glycocalyx engineering method and synthetic mucin panel. **(A)** Glycocalyx engineering by insertion of cholesterylamide-terminal synMUCs into the membranes of live cells. **(B)** Chemical structures and cartoon representations of synMUC panel used in this study, and chemical structure of cholesterylamide (CholA) hydrophobic anchor.

Cell-surface engineering has been accomplished using a variety of molecules from DNA and proteins to polymers and polysaccharides (Lee et al., 2018; Liu, He and Liu, 2019; Zhang, Zhou and Qian, 2021). Methods of attachment have included genetic engineering, covalent conjugation, electrostatic interaction, and hydrophobic insertion. The passive insertion of hydrophobic moieties into the cellular lipid membrane has emerged as one of the most straightforward methods of cell-surface modification. The hydrophobic group is conjugated to the termini of the target group and the cellular growth medium is supplemented with the conjugate. After an incubation period, the cells are pelleted, washed, and then allowed to resume their normal growth conditions Figure 1A. This straightforward approach has been utilized to decorate cells with a plethora of molecules including therapeutic small molecules (Boonyarattanakalin et al., 2004, 2007), peptides (Boonyarattanakalin et al., 2006; Stuhr-Hansen, Vagianou and Blixt, 2017), and polymers (Godula et al., 2009; Mauris et al., 2013; Paszek et al., 2014; Liu et al., 2016; Church and Pokorski, 2020; Delaveris et al., 2021; Naticchia et al., 2021).

Researchers have explored a variety of hydrophobic groups for their efficiency of incorporation into the membrane, as well as their persistence times. In a seminal work probing membrane incorporation of methyl vinyl ketone (MVK) glycopolymers, Woods et al. examined a panel of phosphoglycerolipids and two cholesterol structures (Woods et al., 2015). Woods and others (Hudak et al., 2014) found that phosphoglycerolipids have half-lives of only 4–7 h, however cholesterol structures fair better. One such cholesterol structure, 3β-cholesterylamine (CholA, Figure 1B), had previously been reported by Peterson and co-workers to result in exceptionally long membrane residence times for small-molecule conjugates due to the unique ability to continuously recycle from an endocytic compartment (Boonyarattanakalin et al., 2004; Hymel and Peterson, 2012). Woods et al. corroborated this effect for the MVK polymers, which can persist for many days. Similarly, our own lab found that very high molecular weight glycopolypeptide bottlebrush structures can also recycle to give extended surface residence times (Clauss et al., 2021). Later, Sun and co-workers compared phosphatidylethanolamine- and cholesterol-terminal polyethyleneglycol (PEG) for their membrane incorporation efficiencies, and found the cholesterol anchor was more efficient and longer lasting (Vabbilisetty et al., 2018). CholA has emerged as a premier hydrophobic membrane anchor and has recently been utilized by several labs, including our own, for glycocalyx engineering studies (Honigfort et al., 2019; De Coen et al., 2020; Delaveris et al., 2020; Clauss et al., 2021) Unmodified-cholesterol-terminal polymers have also been used in many recent cell-surface engineering applications (Jia et al., 2017; Uvyn et al., 2019; De Vrieze et al., 2020; Misiak et al., 2020; Wan et al., 2021).

Considering the growing application of CholA in engineering the glycocalyx with precisely defined molecules, we sought to better define the characteristics of this approach. In particular, there is a dearth of information regarding differences in glycocalyx engineering, if any, considering the identity and structure of the molecule to be incorporated, as well as any differences in cell types. Here, we examined the density of membrane incorporation, cell-surface residence time, cytotoxicity, as well as the ability to recycle from endosomes and to be transferred to daughter cells. We compared a panel of CholA modified synthetic mucin glycopolypeptides of varied length, stiffness, and glycan density, in a small panel of adherent and non-adherent cell types. Finally, we explored how glycan density and presentation affect the ability of carbohydrate-binding lectin proteins to target and adhere to the glycocalyx.

## Results

### Selection of cell and polymer panel

Using our previously established methods, we synthesized a panel of synthetic mucins (synMUCs) *via* polymerization of amino acid *N*-carboxyanhydrides (NCAs) (Figure 1B) (Kramer et al., 2015; Clauss et al., 2021; Clauss and Kramer, 2021; Detwiler et al., 2021). Though a variety of molecules have been explored as artificial mucins, we chose our structures due to the fact they are composed entirely of amino acids and sugars found in mucin glycoproteins, contain native linkages, and take on the natural polyproline (PP) II helical conformation that is characteristic of mucins (Kramer et al., 2015; Detwiler et al., 2021; Deleray and Kramer, 2022). The NCA method allows precise tuning of molecular weight (MW) and glycan density, and we can even grow long, glycosylated side-chains from the polypeptide backbone. The long sidechains are attractive since they resemble the bottle-brush structure of mucins that bear longer glycan chains (up to 15 residues) or can even mimic cell-surface proteoglycans that bear glycan sidechains of 100 + residues in length. We utilized our previously developed CholA-functionalized initiators for NCA polymerization, which ensures every synMUC has a single CholA at its termini (Clauss et al., 2021). We used the peptide amino-terminus to conjugate *N*-hydroxysuccinimide (NHS) fluorophores that allowed tracking of the synMUCs. We used NHS-AZ594 in our studies. Conjugation efficiency was determined using the fluorophore absorption relative to synMUC concentration and all data is normalized to account for any differences in fluorophore labeling fraction.

We considered that synMUC length and glycan density, which are properties of great interest in glycocalyx research, could affect incorporation efficiency. Glycan density has been shown to have profound effects on peptide backbone conformation, stiffness, and persistence length. Such factors could affect the ability of macromolecules to pack onto the cell surface. For the panel, we chose a set of three lengths (26, 92, and 217 residues) all with 50% glycan density. Native mucin glycan density (i.e. number of glycosylated residues out of the total mucin domain residues) ranges from ca. 20% up to 70% (UniProt Consortium, 2018), so we selected 50% as a moderate density. To further probe the effect of glycan density and molecular stiffness, we examined synMUCs of two lengths (100 and 177 residues) with 100% glycosylation density. To push the limits of the system further and determine if the presence of branched sidechains would limit glycocalyx engineering, we included a glycobrush structure (Clauss et al., 2021). The brush contained 50 residues in its peptide backbone with 10 residues containing a chain of 13 GalNAcSer residues. The other 40 residues in the backbone were glutamic acid. We selected this composition since native glycocalyx mucin 1 has 20% glycan density on its peptide backbone and can have up to 15 sugars on its sidechain (Hattrup and Gendler, 2008). Additionally, the brush has a total of 130 αGalNAc residues which is in the middle of the range of the number of sugars presented linearly on our other structures. A table of the molar masses and number of sugar units of each structure can be found in the Supplementary Table S1.

In addition to the variety of synMUCs, we included two non-glycosylated structures in our panel, polyproline (PPro) 90mer and polysarcosine (PSar) 100mer. PPro was selected since it adopts the stiff PPII helical structure of mucins but does not bear glycans (Detwiler et al., 2021). PSar was selected since, like PPro, it is hydrophilic and non-ionic (Clauss and Kramer, 2021). However, PSar is disordered and flexible in conformation. Sar is an endogenous non-proteinogenic amino acid and PSar is emerging as a substitute for the widely used polymer PEG (Hu et al., 2018). PSar and PP provide a nice comparison of how backbone conformation affects glycocalyx engineering properties independent of potential interactions with bioactive sugars.

We selected a small panel of adherent and nonadherent cell lines to be coated with the CholA-synMUCs. For adherent cells, we chose human embryonic kidney 293 cells (HEK), Henrietta Lacks (HeLa) cervical cancer cells since they are both widely used for research purposes and have differing native glycocalyces. HEK cells have no reported cell-surface mucins and low chondroitin expression while HeLa cells have a mucin-rich glycocalyx (Thul and Lindskog, 2018). These cell lines therefore present the opportunity to assess how the existing glycocalyx affects the incorporation of additional glyco-macromolecules. We also selected Vero cells, which are derived from monkey kidney epithelia, as a non-human comparison. For suspension cells lines we chose Jurkat cells, which are a human T lymphocyte leukemia line with no reported cell-surface mucins (Thul and Lindskog, 2018), and Raji cells, which are human B lymphocytes of lymphoma origin that express mucins, though at lower levels than HeLa cells (Rouillard et al., 2016).

### Incorporation efficiency of various synMUC structures in adherent vs. suspension cells

**FIGURE 2:**
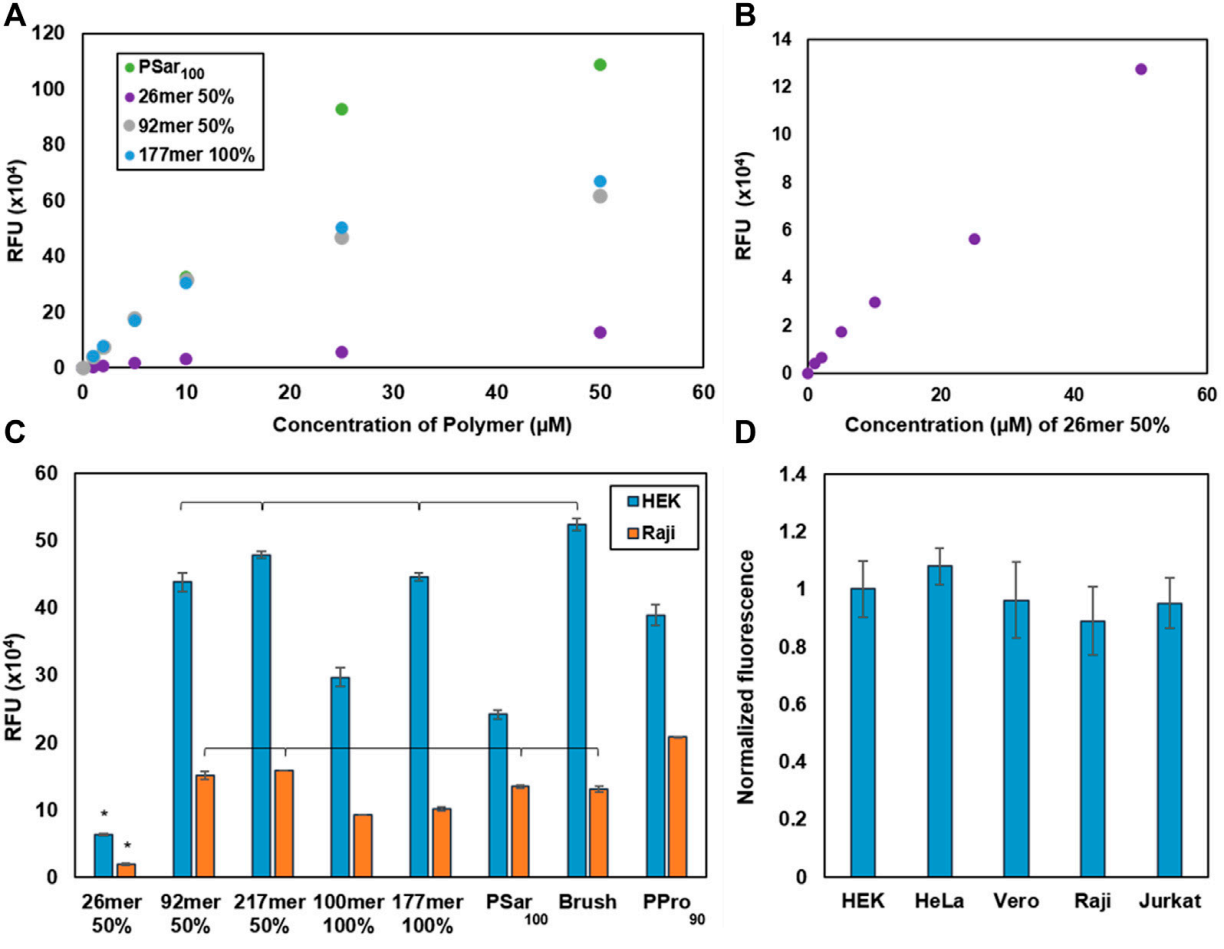
Glycocalyx engineering data to determine saturation concentrations and engineering efficiency by cell type. **(A)** Determination of the membrane saturation concentration in HEK cells for a subset of our synMUC panel. **(B)** Alternate view of the data in **(A)** where we observed no saturation point for the lowest MW synMUC. **(C)** Glycocalyx engineering with a panel of synMUCs of varied composition in adherent (HEK) and suspension (Raji) cells. **(D)** Glycocalyx engineering with the 50% glycosylated 92mer synMUC in three adherent (HEK, HeLa, Vero) and two suspension (Raji, Jurkat) cell lines. Data has been normalized for cell volume. Data in **(A–D)** was collected by flow cytometry immediately after the glycocalyx engineering process. Data in **(C,D)** are averages of medians calculated after gating for live, singlet cells and associated standard error, and were collected in duplicate. *p* < 0.01 is significant; brackets indicate non-significance.

We first sought to determine the synMUC solution concentration that results in saturation and maximum membrane concentration. In brief, adherent HEK cells were lifted into suspension *via* trypsinization, pelleted and resuspended in cellular growth media supplemented with AZDye 594 labeled CholA-synMUCs at concentrations ranging from 1 to 50 µM. Cells were incubated with the CholA-synMUCs (26mer and 92mer 50% glycosylated, 177mer 100% glycosylated, and PSar_100_) for 1 hour at 25°C. After the incubation period cells were pelleted, washed with phosphate buffered saline (PBS) and fluorophore density was analyzed by flow cytometry (FC). We found that aside from the 26mer, the synMUCs displayed a linear increase in density until ca. 10 µM where the concentration began to plateau (Figure 2A). The more flexible PSar_100_ achieved similar cell-surface concentrations at 10 µM but began to plateau at 25 µM. The 26mer was unique in that density increased linearly over the entire range tested and overall cell-surface concentration was lower as compared to the higher MW structures (Figure 2B). It appeared the low MW structure does not incorporate into the membrane as efficiently and that much higher incubation concentrations are needed to achieve the same density as higher MW structures.

The low MW synMUC could simply be less efficiently incorporated in the incubation step. However, considering the reduced steric demands of the lower MW synMUC, we were surprised and hypothesized that after similar membrane incorporation as the other synMUCs it was actually more easily dislodged in the wash step. Polymer loss during cell washes has been observed in similar scenarios (Woods et al., 2015). To probe this hypothesis, we examined the polymer concentration on the cell surface after various numbers of wash steps (see Supplementary Figure S18A) and compared values for the 26mer vs. the 92mer. Indeed, we observed that 52% of the 26mer was lost with the first wash as compared to essentially no polymer loss for the 92mer (see Supplementary Figure S18B). With a second wash, both coatings lost 10%–20% of the synMUC but stabilized with a third wash. We also considered that short polymers could form micelles more efficiently than longer polymers. In this case of an equilibrium of membrane bound vs. solution phase polymers, the equilibrium would be driven forward as shorter polymers were consumed by micelle formation. However, dynamic light scattering experiments indicated this is not the case since both the 26mer and 92mer form a similar distribution of particles in solution (Supplementary Figure S20). Considering this picture, we believe the short synMUCs are simply lost due to the law of mass action.

For proceeding experiments, we chose 10 µM synMUC as our standard glycocalyx engineering concentration since this nicely balances high membrane incorporation with polymer economy. Next, we examined the effect of synMUC structure on membrane incorporation efficiency. We used the coating procedure described above and cells were subsequently examined by FC. Experiments were generally run in duplicate and statistical significance was determined by one-way ANOVA and Tukey tests.

We engineered the glycocalyx of HEK and Raji cells with the full panel of synMUC structures shown in Figure 1B. Remarkably, in both cell lines the incorporation efficiency was not strongly affected by chain length, glycan density, or polypeptide stiffness (Figure 2C). Even the massive bottlebrush structure incorporated at relatively similar concentrations to other structures. The one exception was that, as noted above, the very low MW synMUC 26mer had low incorporation efficiency in both HEK and Raji cells, due to wash loss. Upon first examination it appeared that the incorporation efficiency was lower overall for the Raji suspension cells as compared to adherent HEK cells. However, HEK cells have a significantly larger cell volume than Raji cells which could skew the fluorescence per cell. Indeed, when we normalized the data for cell volume, which was converted from surface area data obtained *via* microscopy, the difference in engineered glycocalyx density was negligible (Figure 2D). Raw, unnormalized data and data normalized to surface area can be found in the Supplementary Figure S12.

To see if the incorporation density is consistent over other adherent and suspension cell lines, we selected a synMUC of medium length and glycosylation density (92mer, 50% glycosylated) for incorporation into the membranes of HEK, HeLa, Vero, Raji, and Jurkat cells. We again noted differences in relative cell size, so we normalized this data for cell volume. Compared side-by-side, the adherent and suspension cells all showed very similar glycocalyx engineering susceptibilities (Figure 2D).

### SynMUC membrane residence time in adherent vs. suspension cells

**FIGURE 3:**
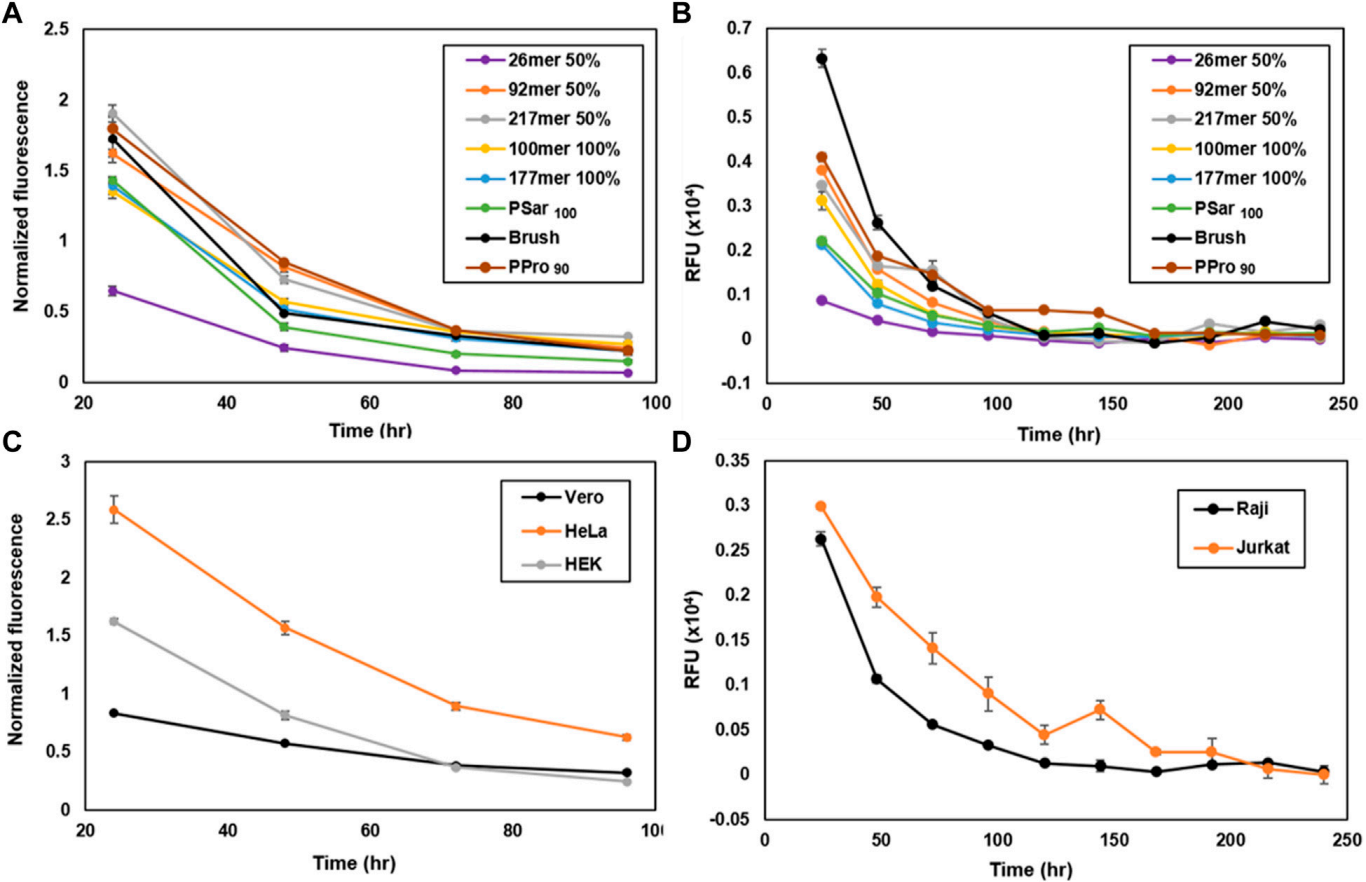
Engineered glycocalyx persistence time considering various synMUC structures and a variety of cell types. Persistence of the synMUC panel in **(A)** HEK cells and **(B)** Raji cells. Data in **(A)** was collected *via* fluorescence microscopy, analyzed using ImageJ software, averaged with standard error for n = 2, and normalized against brightfield images to account for cell density. Data in **(B)** was collected *via* flow cytometry. Data are averages of medians calculated after gating for live, singlet cells and associated standard error, and were collected in duplicate. **(C)** Persistence of 50% glycosylated 92mer synMUC in three adherent (HEK, HeLa, Vero) cell lines. Data was collected as noted for **(A)**. **(D)** Persistence of 50% glycosylated 92mer synMUC in two suspension (Raji, Jurkat) cell lines. Data was collected as noted for **(B)**.

Considering that in some cases it may be desirable to examine the biological effects of the engineered glycocalyx over extended time periods, we wondered if synMUC structure or cell type could affect membrane residence time. We coated Raji and HEK cells with the synMUC panel as previously described above, with the modification that after the PBS wash, cells were subjected to their normal growth conditions and analyzed at various time points. Fluorescence intensity was determined by microscopy for HEK cells and by FC for Raji cells (Figures 3A,B, natural log plots can be found in the Supplementary Figures S2B, S5B, S6). All fluorescence data collected *via* imaging was normalized against the number of cells present. SynMUC density dropped rapidly in the first 2 days post-coating in both cells lines and then reached near constant concentration. Remarkably, on Raji cells the glycocalyx could be detected for up to 10 days for all structures except the 26mer. On HEK cells, the coating lasted for 4 days. HEK cells could not be examined for longer due to reaching confluency during the experiment period. Table 1 shows the calculated half-lives for each structure and cell type. For HEK cells, the half-lives ranged from ca. 20–28 h. The lower MW weight synMUCs and the more flexible polySar were lost closer to 20 h while the longer and stiffer structures persisted longer. In Raji cells, the half-lives of the engineered glycocalyces were ca. 36–62 h. Again, suspension cells can be examined for longer time periods than adherent cells that become confluent. We observed a similar trend where the lower MW synMUCs were lost more rapidly than the higher MW structures. In both cases the trend is not precisely correlated with structure MW indicating other factors are at play.

**TABLE 1:** Calculated half-lives for the synMUC panel in a variety of adherent and suspension cell lines. Data used in these calculations is shown in Figure 3.

synMUC	Cell line	T_1/2_ in hours
26mer 50%	Raji	36.0
92mer 50%	Raji	38.0
217mer 50%	Raji	61.5
100mer 100%	Raji	59.8
177mer 100%	Raji	49.5
Brush	Raji	40.8
PSar_100_	Raji	53.7
PPro_90_	Raji	38.5
26mer 50%	HEK	20.4
92mer 50%	HEK	22.7
217mer 50%	HEK	24.7
100mer 100%	HEK	26.6
177mer 100%	HEK	27.7
Brush	HEK	25.7
PSar_100_	HEK	20.8
PPro_90_	HEK	23.9
92mer 50%	Jurkat	38.5
92mer 50%	HeLa	34.7
92mer 50%	Vero	49.5

To confirm that this behavior is not unique to HEK and Raji cells, we performed the same experiment using the 50% glycosylated 92mer synMUC on HeLa, Vero, and Jurkat cells side-by-side with HEK and Raji cells (Figures 3C,D). Again, the coating density dropped within the first 1–2 days post-coating and then reached a more stable population with slow decline. The suspension cell half-lives were essentially identical at ca. 38 h (Table 1). The adherent cell glycocalyx half-lives for this structure ranged from ca. 23 h in HEK cells and 35 h in HeLa cells, to a remarkable 50 h in Vero cells. Considering that the initial coating density for this synMUC was nearly identical for these cells, this cannot account for the differences. The growth rates of these cell lines also fail to explain the differences, as they all have similar reported doubling times at 20–24 h (Nahapetian et al., 1986; Tani et al., 1996; Schoene and Kamara, 1999; Cervera et al., 2011). Based on prior reports (Boonyarattanakalin et al., 2004; Hymel and Peterson, 2012; Woods et al., 2015), we presume that during the first 24 h post-engineering, a fraction of the cell-surface synMUC is incorporated into endosomes that can recycle material back to the cell surface for many days. Assuming this process occurs for our synMUCs, we speculate that differences in the internalization and recycling process could be a factor in the glycocalyx half-lives.

### Endosomal pooling of synMUCs and subsequent transfer to daughter cells

**FIGURE 4:**
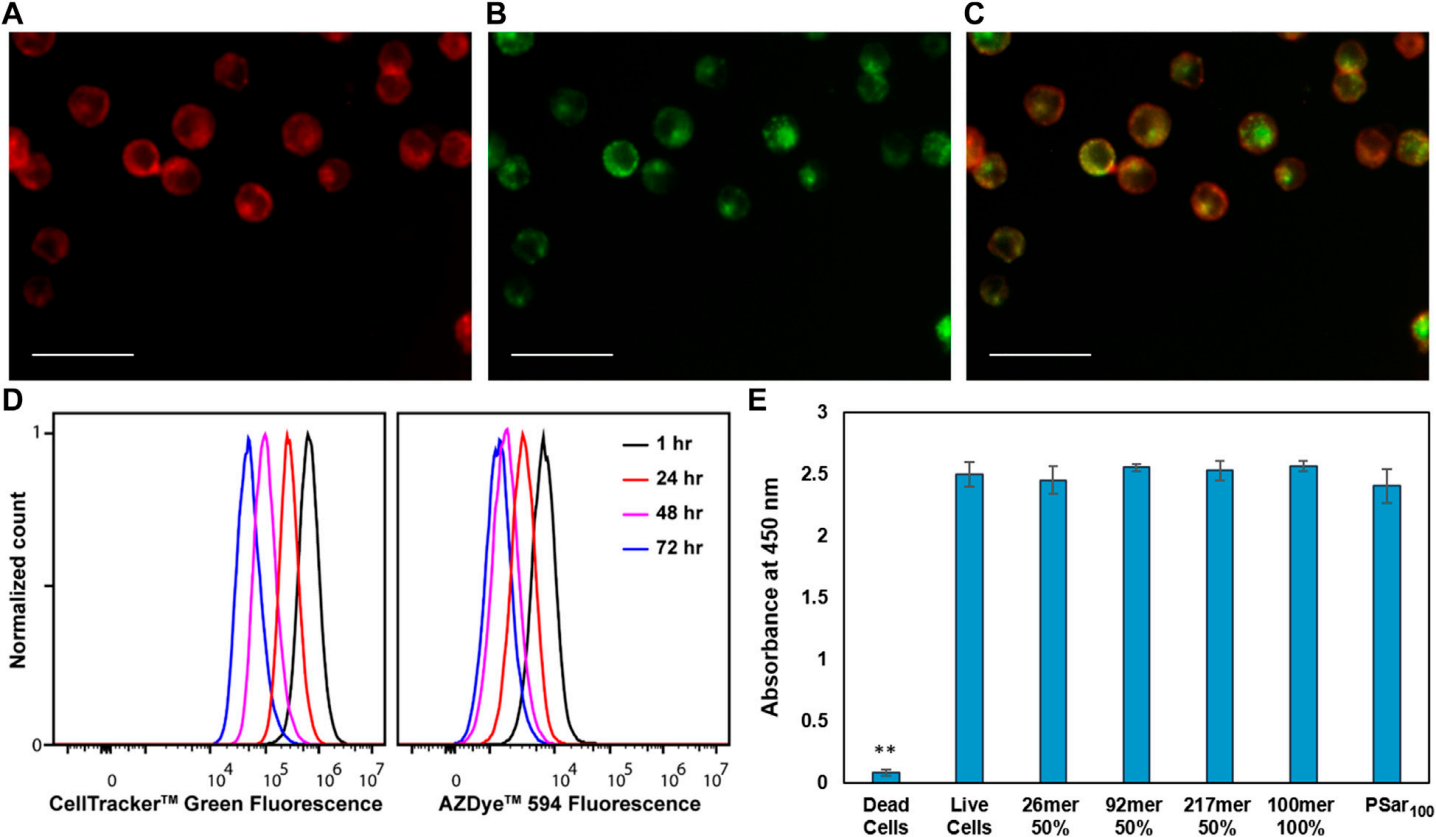
CholA-synMUCs are non-cytotoxic, are internalized into recycling endosomes, and are transferred to daughter cells. Fluorescence images demonstrating co-localization of **(A)** AZ594-labeled 50% glycosylated 92mer synMUC with **(B)** CF488A-transferrin in HEK cells. **(C)** is an overlay of the images in **(A,B)**. Scale bars for **(A**–**C)** are 50 µm. **(D)** Flow cytometry histogram data collected over period of 4 days for Raji cells coated with 50% glycosylated 92mer synMUC and treated with Cell Tracker green. Cells were analyzed at various time points. Unimodal populations were observed suggesting equal distribution of polymer among daughter cells. **(E)** CCK8 Cytotoxicity assay for HEK cells glycocalyx engineered with various synMUCs. Data was collected 4 days post-engineering. No statistical effect on viability was observed as compared to the live cell control. ** Indicates statistical significance.

To confirm the presumption that the synMUCs behave similarly to other CholA conjugates and pool into recycling endosomes, we performed colocalization experiments of our AZ594-labeled 50% glycosylated 92mer synMUC with CF488A-labelled transferrin. Transferrin is known to traffic to early endosomes and endocytotic recycling compartments (Mayle et al., 2012). HEK and Raji cells were treated with the synMUC for 60 min as previously described except that 30 min into the procedure, the CF488A-transferrin was added. The cells were washed, fixed, and then imaged. As expected, synMUC nicely co-localized with the CF488A-transferrin, indicating that these structures indeed pool in endosomes and later recycle to the cell surface. Figures 4A–C shows images of HEK cells and data for Raji cells can be found in the Supplementary Figure S14. We previously demonstrated that, despite its large hydrodynamic volume, the glycobrush can also be internalized and recycled (Clauss et al., 2021). To estimate the fraction of polymer resident in the cell membrane as compared to internalized polymer, we quantified the fluorescence colocalization of the 50% glycosylated 92mer and transferrin for both HEK and Raji cells (See Supplementary Figure S13). We found that the cell surface fraction was 45 ± 10% for HEK and 39 ± 11% for Raji cells. This method likely underestimates the cell surface fractions considering that in some areas the membrane will overlay with internal endosomes. Another method that has been employed utilized biotin-capped CholA-terminal polymers and membrane impermeable anti-biotin antibodies with similar results (Woods et al., 2015).

Previous reports indicated that CholA-conjugates can be transferred to daughter cells upon cell division (Woods et al., 2015). To confirm our synMUCs behave similarly, we utilized CellTrackerdyes, which pass through the membrane of live cells where they fluoresce. The dyes can pass to daughter cells, but not adjacent cells, which allowed us to track our synMUCs as they are transferred during cell division. HEK and Raji cells were treated simultaneously with the 92mer 50% glycosylated synMUC and CellTracker, washed, and allowed to grow as normal. FC analysis was performed daily over 4 days. Data for Raji cells is shown in Figure 4D while imaging data for HEK cells can be found in the Supplementary Figure S14. As shown in Figure 4D, the intensity of both fluorophores dims in the cell population over the duration of the experiment and no divergent populations are observed, thus indicating uniform transfer of the synMUC engineered glycocalyx to progeny cells.

### Cytotoxicity of engineered glycocalyces

Considering that our synMUCs are composed entirely of natural amino acids and glycans in native mucin conformations, we would not predict general cytotoxicity to be associated with these molecules. Prior work on our synMUCs as free molecules dissolved in growth media has indeed indicated they are well tolerated in a variety of cell lines (Kramer et al., 2015; Zhou et al., 2018; Clauss et al., 2021). However, we sought to confirm that the CholA glycocalyx engineering strategy itself did not cause toxicity issues over time. Using CCK-8 assays, we were pleased to confirm that 4 days post-engineering of the glycocalyx of HEK cells no statistically significant change in viability was detected for any of the structures examined in our panel (Figure 4E) Similarly, no change in the viability of Raji cells was detected by FC for 10 days post-engineering (Supplementary Figures S16, S17).

### Lectin-synMUC interactions

Lectins are a class of carbohydrate-binding proteins that play important roles in human health and disease (Lam and Ng, 2011). Many lectins have multiple carbohydrate recognition domains and as such can be sensitive to the density of glycan presentation. Additionally, prior work on synthetic polymers bearing sugars has indicated that molecular crowding can interfere with binding and that optimal glycan spacing may exist to balance accessibility with maximal binding (Cairo et al., 2002; Godula and Bertozzi, 2012; Tian et al., 2012; Kiessling and Grim, 2013). In proof-of-concept experiments to highlight the utility of this glycocalyx engineering strategy, we treated Raji cells with designed glycocalyces with the lectin Helix pomatia agglutinin (HPA), which is known to bind αGalNAc (Sanchez et al., 2006). We used PPro_90_ and PSar_100_ as non-glycosylated controls. HPA-fluorescein was used for direct visualization of binding by FC.

**FIGURE 5:**
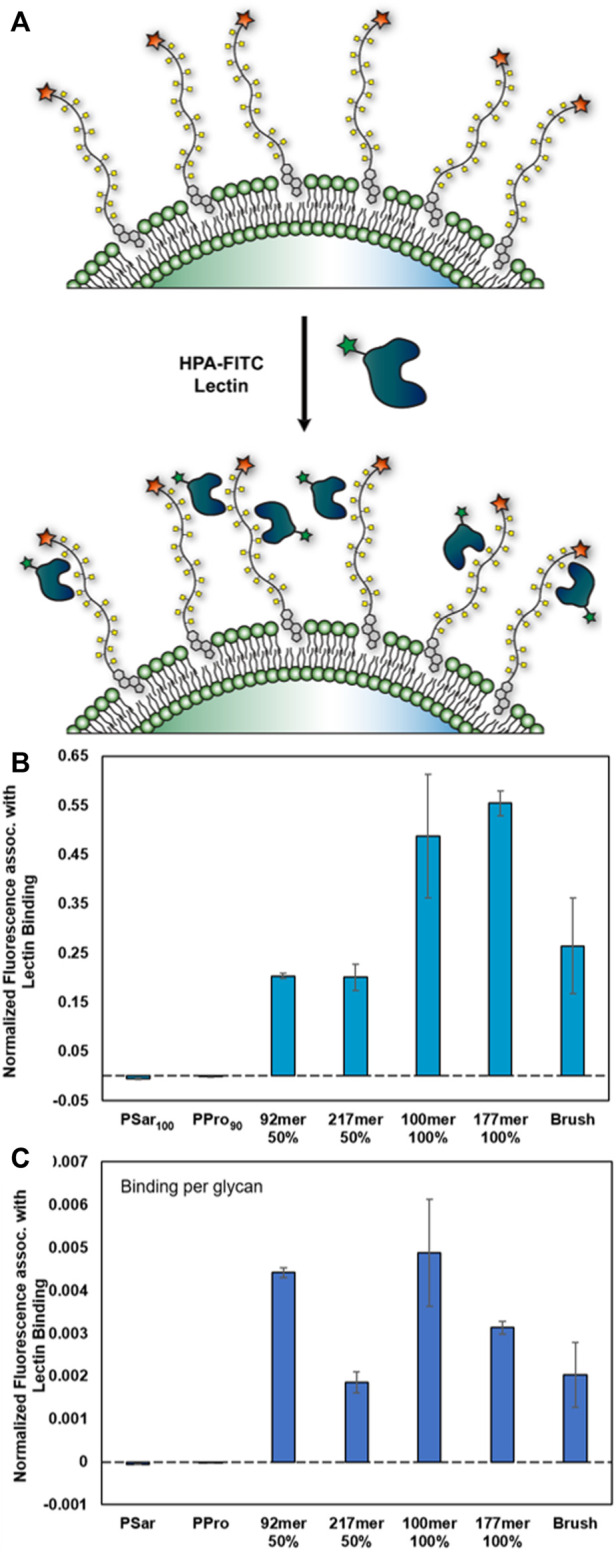
Binding of lectin HPA-fluorescein conjugate to engineered glycocalyces of varied composition **(A)** Cartoon representation of αGalNAcSer binding by hexameric HPA lectin. **(B,C)** Data were collected in Raji cells *via* flow cytometry and are averages of medians calculated after gating for live, singlet cells and associated standard error, and were collected in duplicate. Data has been normalized against background binding and to account for differences in cell-surface density. **(B)** Binding of HPA to synMUC engineered glycocalyces. **(C)** Binding of HPA on a per-residue basis.

We observed HPA binding to all structures bearing αGalNAc and, as expected, we saw no binding to PSar and PPro (Figure 5). Data were normalized to account for any differences in glycocalyx coating density. From the direct analysis of the FC data, we noted higher lectin binding for the 100% glycosylated structures as compared to the 50% glycosylated synMUCs (Figure 5A), but lower binding for the brush. However, when reanalyzing the data in terms of binding-per-glycan we observed that HPA had lower affinity for the longer chain lengths and the brush (Figure 5B). For example, the binding efficacy of the 50% glycosylated 217mer was less than half of that of the 92mer. Similarly, the binding of HPA to the 100% glycosylated 100mer was higher than that of the 177mer or the brush. We did examine lectin binding to a 26mer 50% glycosylated glycocalyx and this can be found in the Supplementary Figure S19. However, considering the substantial difference in surface density we do not believe the data can be directly compared to the other structures.

## Discussion

Study of the cellular glycocalyx is a rapidly growing area in need of tools to precisely probe molecular structure-function relationships. Cell-surface engineering with synthetic mucin-mimetic materials has emerged as a strategy which has already provided insights on glycocalyx mechanics, cancer biology, and infection (Paszek et al., 2014; Huang et al., 2017; Delaveris et al., 2020; Honigfort et al., 2021). Though various materials have been used as mucin mimics, a common engineering strategy has been used. Mimics terminated in hydrophobic anchors passively insert into the membranes of live cells when supplemented into the cellular growth medium. This method is simple, accessible, and does not require genetic engineering nor addition of enzymes or chemical conjugation reagents. The CholA group has risen to the top of the hydrophobic anchor pool due to its superior residence time as compared to diacylphospholipids. Despite the popularity of this glycocalyx engineering strategy, there is a dearth of information about how variation in mucin-mimetic structure affects membrane incorporation and residence time, or if the strategy is comparable across cell lines.

To address these questions, we engineered the glycocalyces of a variety of cell lines with a panel of synMUC structure variants of different lengths, glycosylation densities, and conformations. For the mucin mimics, we utilized in-house prepared polypeptides bearing mucin glycans. These materials are composed entirely of authentic mucin sugars and amino acids in their native linkages, and they adopt the PPII conformation of the mucin backbone. To examine the role of structure and conformation separate from glycosylation, we compared membrane incorporation of PPII helical PPro and flexible, disordered PSar.

With one exception, the entire CholA-terminal synMUC panel was found to efficiently incorporate into both suspension and adherent cell membranes with similar densities. The outlier was the lowest MW synMUC with only 26 residues. This was surprising since we would assume a lower steric barrier for the 26mer as compared to our much higher MW structures such as the 217mer or the brush. However, based on examining the effect of the wash step, we believe the low MW synMUCs are more readily lost by agitation of the membrane and likely do incorporate similarly to the others. These data were unexpected since Godula et al. reported higher efficiency incorporation for a 30mer glucosylated PEG vs. a 140mer or 440mer (Honigfort et al., 2021). The discrepancy could potentially be due to the polymer structure, which in our case is inherently more rigid, the cell type employed, which in their case was red blood cells which have a compact and minimal glycocalyx and do not endocytose, or the structural differences in the cholesterol structures used. Additionally, it was not noted that the data was normalized for any differences in fluorophore labeling. In any case, the lower density of the 26mer synMUC could simply be overcome by incubating with a higher concentration during the initial engineering process. We determined the saturation concentration of the synMUCs on the cell surface, which was similar for all structures besides the 26mer, and utilized 10 µM for all further experiments.

Similar to prior reports on other CholA-conjugates (Boonyarattanakalin et al., 2004; Hymel and Peterson, 2012; Woods et al., 2015), we validated our synMUCs are internalized into recycling endosomes and transferred to daughter cells. The recycling process allows the engineered glycocalyces to persist on adherent cells for up to 4 days and suspension cells for up to 10 days. Adherent cells become confluent within ca. 4 days and cannot be examined longer. We did observe variation in the half-lives of individual polymers by structure and by cell type. We speculate this is due to variations in the endocytic internalization and recycling processes or potentially variations in native membrane composition or glycocalyx structure. Generally, the half-lives of lower MW synMUCs were shorter. For all structures, we saw a rapid decline in surface polymer over ca. the first day, followed by a slow and steady decline over the remainder of the experiment. Prior work by Woods et al. using glycosylated MVK showed similar trends in membrane residence time. The rapid initial loss is likely due to the internalization process. No cytotoxicity was observed for any engineered glycocalyx structure in HEK cells over the 4-day period or for Raji cells over the 10-day period.

Finally, we examined binding of lectin HPA, which has specificity for αGalNAc(Sanchez et al., 2006), to the engineered glycocalyces of Raji cells. Interestingly, we saw that binding was more strongly influenced by chain length than by glycan density. For example, structures of similar length (92mer, 100 mer) but 50% vs. 100% glycosylation density had similar per-residue binding. By contrast, structures with identical glycosylation density (50% or 100%) but differing chain lengths (92mer vs. 217mer, or 100mer vs. 177mer) had very different per-residue binding where the affinity for the shorter polymers was higher. Prior work has indicated that spacing of glycan residues along the backbone affects affinity, where crowding due to high glycan density inhibited binding (Cairo et al., 2002; Kiessling and Grim, 2013). Therefore, we were surprised that HPA binding to the 100% glycosylated 100mer was essentially double that of the 50% glycosylated 92mer and that their per-residue binding was comparable. It is notable however, that glycan-lectin binding studies are typically performed by precipitation assays or by immobilizing materials on a surface. Here, we are displaying the structures in their native environment with the innate ability to mobilize around the membrane. Our data indicate that glycocalyx organization and diffusion of binding molecules through the glycocalyx could be a factor in such affinity interactions. Prior work has shown that mucins, and analogs, cluster and organize in the membrane and that this is affected by their molecular size (Paszek et al., 2014). It is also worth considering that HPA is hexameric and could induce clustering, this affecting diffusion and accessibility for further binding events. We have normalized data to account for background binding, and we saw no effect on cells engineered with structures lacking αGalNAc (polyPro, polySar), so we believe the effects are specific to αGalNAc in the engineered glycocalyx.

## Conclusion

Studies of the glycocalyx have been particularly challenging due to the inherent heterogeneity of its component structures and lack of tools to modify it with precision. Here, we present data indicating that the use of CholA-terminal synthetic mucins is a simple and robust method of precision glycocalyx engineering. The method allows materials of diverse structural identity to be applied to the cell surface, is not associated with any toxicity or ill effects, and results in a long-lasting glycocalyx enabling many days of experiments. The workflow of the glycocalyx engineering method is exceptionally simple, which will allow access to diverse glycobiology studies in labs worldwide.

## Materials and methods

### Synthetic mucin preparation

All synthetic mucins were prepared in accordance with previous literature. See Supplementary Material for more detail.

### Cell culture

Adherent cells lines (Human embryonic kidney (HEK) 293, HeLa, and Vero E6 primate cells) were maintained in high glucose Dulbecco’s modified Eagle medium supplemented with 10% (v/v) FBS, 2 mM l-glutamine, and 1% penicillin/streptomycin. Suspension cells lines (Raji B-lymphocytes and Jurkat T-lymphocytes) were maintained in RPMI-1640 medium (ATCC 30-2001) supplemented with 10% (v/v) FBS, 2 mM l-glutamine, and 1% penicillin/streptomycin. All cells were grown at 37°C with 5% CO_2_.

### General glycocalyx engineering procedure

AZDye 594-labeled polymers were dissolved in complete cellular growth media at 10 µM and sterile filtered through a 0.2 µm membrane. If applicable, cells were trypsinized and neutralized with complete media according to ATCC guidelines. Cells were pelleted by centrifugation at 100 × g for 5 min. Cells were suspended in polymer-free media as a mock-engineered control or media containing polymer at 10^6^ cells/mL for downstream imaging or 10^7^ cells/mL for analysis *via* flow cytometry. Cells were incubated with polymer at room temperature for 1 hour. Post-incubation, cells were pelleted, washed with PBS, and either resuspended in PBS for immediate flow analysis or in complete media for cell expansion. To examine persistence, expanded cells were imaged on a brightfield/fluorescent microscope or analyzed *via* flow cytometry every 24 h. Flow cytometry data was acquired with at least 10,000 events for all samples, and 0.1 μg/ml DAPI was added as a live/dead discriminator. Data was analyzed with either ImageJ or Flow Jo and was normalized and plotted in Excel. For persistence data, an exponential decay fit was applied and used to estimate the half-life. See Supplementary Material for more information on controls, nuclear staining, flow cytometry gating, and data analysis.

### CCK-8 cytotoxicity assay

Polymer-coated HEK 293 cells were analyzed *via* CCK-8 assay 4 days after polymer incubation. Positive/live control cells were prepared at time of incubation. One hour prior to assay, a negative/dead cell control was prepared by treating cells with 1% Triton X-100 in complete media. Following this, 50 µL/well CCK-8 reagent was added to cells and also to media as a blank. Cells were incubated with reagent for 3 h at 37 °C, and absorbance was measured at 450 nm. Data were collected in triplicate, normalized against the blank, averaged, and plotted with associated standard deviation. An ANOVA and Tukey-test were conducted.

### Evaluating mechanisms of polymer longevity

To track endocytic recycling, a transferrin colocalization assay was conducted. In brief, HEK cells were coated with 10 μM AZDye 594-50% glycosylated 92mer in serum-free media. CF488A-transferrin was added to a final concentration of 30 μg/ml for the last 30 min of a 1-h incubation with polymer. Media was exchanged for complete media, and cells were incubated at 37°C for 15 min to allow for transferrin trafficking. Cells were washed with PBS, fixed, and fluorescently imaged. Similarly, a CellTracker assay was used to evaluate polymer distribution among daughter cells. Raji cells were incubated at 10^7^cells/mL with 10 µM AZDye 594-50% glycosylated 92mer and 5 µM CellTracker™ Green CMFDA (Thermo C2925) in serum-free media for 1-h at room temperature. Post-incubation, cells were washed with PBS, resuspended in complete media, and plated. At 1, 24, 48, and 72 h post-incubation, 1⋅10^6^ cells/sample were pelleted, washed once with PBS, resuspended in PBS, and analyzed *via* flow cytometry. The distribution of polymer to daughter cells was evaluated *via* population comparison in FlowJo. See Supplementary Material for more details along with HEK cell protocol.

### Lectin binding

Raji cells were first incubated at 10^7^cells/mL with 10 µM AZDye 594-labeled polymer panel for 1 hour at room temperature. Post-incubation, cells were washed once with PBS. Polymer-treated cells were then incubated with 5 μg/ml helix pomatia lectin-FITC (EY Labs F-3601-1) in 1% BSA in PBS (Mg^++^ and Ca^++^) on ice for 1 hour. Cells were then washed twice, resuspended in 1% BSA in PBS, and evaluated *via* flow cytometry. Flow analysis was conducted in FlowJo. See Supplementary Material for more detail on flow cytometry acquisition, data analysis, and normalization.

## Data Availability

The original contributions presented in the study are included in the article/Supplementary Material, further inquiries can be directed to the corresponding author.
