# Supplementary material for: Principles of glycocalyx engineering with hydrophobic-anchored synthetic mucins
Source: Front Cell Dev Biol. 2022 Oct 17;10:952931. doi: 10.3389/fcell.2022.952931 (PMC9621330; doi:10.3389/fcell.2022.952931)
Supplement: Supplementary file 1 [file DataSheet1.pdf]

Supplementary Material

Principles of glycocalyx engineering with hydrophobic-anchored synthetic mucins

Casia L. Wardzala[1], Zach S. Clauss[1], and Jessica R. Kramer[1]
[1]Department of Biomedical Engineering, University of Utah, Salt Lake City, UT, USA

* Correspondence: Jessica Kramer, PhD. jessica.kramer@utah.edu

Table of Contents

1 Experimental Procedures 2

1.1 Instrumentation and general methods 2

1.2 Synthetic mucin preparation 3

1.2.1 Fluorophore labeling 3

1.2.2 Fluorophore labeling efficiency determination 3

1.3 Biological procedures 3

1.3.1 Cell culture 3

1.3.2 General glycocalyx engineering procedure for imaging 4

1.3.3 General glycocalyx engineering procedure for flow cytometry 4

1.3.4 CCK-8 cytotoxicity assay 5

1.3.5 Transferrin colocalization study 5

1.3.6 Polymer distribution among daughter cells 5

1.3.7 Lectin binding 5

2 Supplementary Figures and Tables 6

2.1 Supplementary Figures 6

2.2 Supplementary Tables 17

2.3 NMR Spectra 19

3 References 19

1 Experimental Procedures

1.1 Instrumentation and general methods

Infrared spectra were recorded on a Bruker Alpha ATR FT-IR Spectrophotometer. Deionized water (18 MΩ-cm) was obtained by passing in-house deionized water through a Thermo Scientific MicroPure UV/UF purification unit. Size exclusion chromatography with tandem light scattering and refractive index detection (SEC/MALS/RI) was performed on an Agilent 1260 Infinity liquid chromatograph pump equipped with a Wyatt DAWN HELEOS-II light scattering (LS) and a Wyatt Optilab T-rEX refractive index (RI) detectors. Separations were achieved using 10^5, 10^4, and 500 Å Phenomenex Phenogel 5 µm columns using 0.10 M LiBr in DMF as the eluent at 60°C. All SEC/MALS/RI samples were prepared at concentrations of 3 mg/mL. ^{1}H NMR spectra were recorded on a Varian Mercury spectrometer (400 MHz). The hydrodynamic diameters of polymers (unlabeled), prepared at ~ 1 µM in 1X DPBS and filtered through 0.2 µm membrane, were obtained on a Malvern Zetasizer Nano ZS (backscatter at angle of 173°). Cells were imaged on a Laxco LMI-6000 microscope and processed with ImageJ version 1.51j8. Flow cytometry data was acquired on a Beckman Coulter Cytoflex S flow cytometer and analyzed using Flow Jo v10.8.1.

1.2 Synthetic mucin preparation

O-(3,4,6-tri-O-acetyl-2-acetamido-2-deoxy-α-D-galactopyranosyl)-L-serine (αGalNAcSer), L-alanine (Ala), L-glutamic acid γ-tertbutyl ester (*t*BuGlu), *N*ε-(allyloxycarbonyl-L-isoleucyl)-L-lysine (AIK), L-proline (Pro), and sarcosine (Sar) *N*-carboxyanhydrides (NCAs) and resulting polypeptides were synthesized according to previous literature.[1–4] The cholesterolamide functionalized Ni initiator (Ni-CholA) was synthesized as previously described.[2,4] In brief, monomers were mixed with Ni-CholA in varied ratios to form the desired length polypeptide. For 50% glycosylated copolymers, αGalNAcSer, Ala, and *t*BuGlu NCAs were mixed in a ratio of 2:1:1 prior to treatment with Ni-CholA. The glycobrush was prepared by copolymerization of Reactions were monitored by ATR-FTIR and, upon completion, were analyzed by SEC/MALS/RI and ^{1}H NMR. All data was in accordance with previous literature.[1–4] Acetate and tBu protecting groups were removed as previously described.[3] The presence of the CholA group on the polypeptide termini was verified by ^{1}H NMR as shown in the representative spectra. All polymers were purified by precipitation followed by dialysis against MilliQ water in 2000 molecular weight cutoff tubing, or spin filtration using Amicon Ultra-2 or Ultra-15 MWCO 3kDa filters and spun down 5 times, diluting in MilliQ water each time.

1.2.1 Fluorophore labeling

AZDye 594 NHS ester was dissolved at 10 mg/mL in dimethylsulfoxide. Polymer was dissolved at the maximum concentration for solubility in MilliQ water. An aliquot from the polymer stock solution was transferred to a 4 mL vial equipped with a stir bar. A 1 M stock solution of $NaHCO_3$ was used to adjust the polymer solution in the 4 mL vial to a 0.2 M $NaHCO_3$ concentration. A volume of the AZDye 594 NHS ester solution was added to the 4 mL vial corresponding to 5 equivalents of fluorophore per polymer chain. The solution was shielded from light and allowed to react overnight. The following day, unreacted fluorophore and salts were removed by spin filtration using Amicon Ultra-15 MWCO 3kDa filters and spun down 5 times, diluting in MilliQ water each time.

1.2.2 Fluorophore labeling efficiency determination

To determine the fluorophore labeling efficiency, labeled polymers were dissolved at 100 µM in MilliQ water and analyzed on a Nanodrop 2000 Spectrophotometer using the proteins and labels procedure. The spectrophotometer determines the concentration of the selected fluorophore. For example, if the spectrophotometer determines the [AZDye 594] as 55.0 µM, the labeling efficiency would be reported as 55%.

1.3 Biological procedures

1.3.1 Cell culture

Adherent cells lines (Human embryonic kidney (HEK) 293, HeLa, and Vero E6 primate cells) were maintained in high glucose Dulbecco's modified Eagle medium supplemented with 10% (v/v) FBS, 2 mM L-glutamine, and 1% penicillin/streptomycin. Suspension cells lines (Raji B-lymphocytes and Jurkat T-lymphocytes) were maintained in RPMI-1640 medium (ATCC 30-2001) supplemented with 10% (v/v) FBS, 2 mM L-glutamine, and 1% penicillin/streptomycin. All cells were grown at 37°C with 5% CO_2.

1.3.2 General glycocalyx engineering procedure for imaging

All imaging studies were conducted with adherent cell types. First, AZDye 594-labeled polymers were dissolved in complete adherent cell media at 10 µM and sterile filtered through a 0.2 µm membrane. Cells were trypsinized and neutralized with complete media according to ATCC guidelines. 100,000 cells/sample were pelleted by centrifugation at 100 xg for 5 minutes. Cells were suspended at 10^6 cells/mL in polymer-free media as a mock-engineered control or media containing polymer. Cells were incubated, covered, at room temperature for one hour. Post-incubation, cells were pelleted, washed with PBS, resuspended in 500 µL complete media, and plated on a 24-well plate. All cells were left to grow at 37 °C. At timepoints 24, 48, 72, and 96 hours following polymer treatment, cells were imaged with a brightfield/fluorescent microscope. Separate wells of treated and control cells were also nuclear-stained with Hoescht 33342 24 hours post-incubation and imaged. Studies were run in duplicate, with n = 4 for image analysis. Fluorescence and cell margins (from brightfield images) were measured with ImageJ via thresholding and pixel quantification. AZDye™ 594 fluorescence was normalized to # of cells (via cell margin quantification) and plotted against time. An exponential decay fit was applied in Excel and used to estimate the half-life. Natural log plots were also created in Excel to evaluate the exponential fit.

1.3.3 General glycocalyx engineering procedure for flow cytometry

Flow cytometry was used to conduct: 1) concentration studies in HEK 293 cells to determine ability to control cell surface density, 2) persistence studies with suspension cell types (i.e. Raji and Jurkat), 3) density studies analyzed post-polymer incubation with both suspension and adherent cells, and 4) wash studies in Raji cells with 50% glycosylated 26mer and 92mer (see Supplementary Figure 17). AZDye 594-labeled polymers were dissolved in complete media, at 10 µM for most studies, and sterile filtered through a 0.2 µm membrane. To correlate polymer concentration to density on membrane, concentrations of 1, 2, 5, 10, 25, and 50 µM were prepared. At this time, cells were trypsinized if necessary. Cells were pelleted and suspended in media +/- polymer at 10^7 cells/mL. Cells were incubated, covered, at room temperature for one hour. Post-incubation, cells were pelleted, washed with PBS (and washed further for wash studies), and either resuspended in PBS for immediate flow analysis or in complete media for expansion and later flow preparation. To examine persistence, every 24 hours, expanded cells were counted and $0.5\text{-}1{\cdot}10^6$ cells/sample were pelleted, washed once with PBS, and resuspended in PBS for flow analysis. DAPI (0.1 µg/mL from ThermoFisher # 62247) was added as a live/dead discriminator. Flow cytometry data was acquired with at least 10,000 events for all samples. The gating tree was as follows: 1) FSC/SSC to exclude debris, 2) singlet gate (FSC-height vs. FSC-area), 3) live gate (DAPI negative), and 4) SSC/PE-Texas Red (AZDye™ 594 positive), when applicable. See Supplementary Figure 1 for gating example. Data were analyzed by taking median fluorescence intensity (MFI) values, adjusting for fluorophore conjugation efficiency by dividing raw MFI by fractional conjugation efficiency, and averaged. Most data were collected in duplicate, and data variance and significance was analyzed via standard error and p-values calculated by ANOVA and Tukey testing. Exponential decay fits were applied to persistence data and used to estimate the half-life. Natural log plots were also created in Excel to evaluate the exponential fit. To compare density of incorporation across all cell types, RFU data was normalized to the volume of the cell. The surface areas of cells in suspension were quantified in image J. Assuming a spherical shape, volumes (V) were calculated from surface area (SA) via $V = (4/3)*\pi*[\mathrm{sqrt}(SA/\pi)]^3$ *and* normalized against resulting values for HEK 293 cells. Data normalized against surface area is also shown in the SM (Fig 12).

1.3.4 CCK-8 cytotoxicity assay

Polymer-coated HEK 293 cells were analyzed via CCK-8 assay 4 days after polymer incubation. Positive/live control cells were prepared at time of incubation. One hour prior to assay, a negative/dead cell control was prepared by treating cells with 1% Triton X-100 in complete media. Following this, 50 µL/well CCK-8 reagent was added to cells and also to media as a blank (volume of reagent scaled for 24-well plates). Cells were incubated with reagent for 3 hrs at 37°C and absorbance was read at 450 nm on a SpectraMax M2 Microplate Reader. Data were collected in triplicate, normalized against the blank, averaged, and plotted with associated standard deviation. An ANOVA and Tukey-test were conducted.

1.3.5 Transferrin colocalization study

One million cells, either Raji or HEK 293, were coated with 10 µM AZDye™ 594-50% glycosylated 92mer in serum-free media according to above protoco. CF488A-transferrin was added to a final concentration of 30 µg/mL for the last 30 minutes of a 1-hour incubation with polymer. Media was exchanged for complete media, and cells were incubated at 37°C for 15 minutes to allow for transferrin trafficking. Cells were washed with PBS, allowed to sediment onto coverslips at 1·106 cells/mL PBS, fixed with 4% paraformaldehyde, and fluorescently imaged. Transferrin colocalization data was used to estimate the fraction of polymer inserted in cell membrane, as compared to internalized polymer, for both HEK 293 and Raji cells. Fluorescence owing to CF488A-transferrin (F_{488}) excitation and, separately, AZDye™ 594-polymer excitation (F_{594}) was thresholded and pixel-quantified in ImageJ. The percentage of polymer on the cell membrane was calculated by $[(F_{594}-F_{488})/F_{594}]*100\%$ in Excel with an n = 10 per cell line, and averages and standard deviations were also quantified.

1.3.6 Polymer distribution among daughter cells

For this study, suspended Raji cells were analyzed via flow cytometry and adherent HEK 293 via imaging. Raji cells were incubated at 10^7cells/mL with 10 µM AZDye™ 594-50% glycosylated 92mer and 5 µM CellTracker™ Green CMFDA (Thermo C2925) in serum-free media for one-hour at room temperature. Unstained and single-color controls were also prepared at this time. Post-incubation, cells were washed with PBS, resuspended in complete media, and plated. At 1 hr, 24 hr, 48 hr, and 72 hr post-incubation, $1{\cdot}10^6$ cells/sample were pelleted, washed once with PBS, and resuspended in PBS for flow analysis. Flow cytometry data was acquired with at least 10,000 events for all samples. The distribution of polymer to daughter cells was evaluated via population comparison in FlowJo. Similarly, 150,000 adherent HEK 293 cells were incubated with 300 µL 10 µM AZDye™ 594-50% glycosylated 92mer and 25 µM CellTracker™ Blue CMHC (Thermo C2111) in serum-free media for one-hour at room temperature. Polymer-free and unlabeled controls were also prepared at this time. Post-incubation, cells were washed with PBS, plated in complete media, and fluorescently imaged 24 and 48 hours after treatment.

1.3.7 Lectin binding

Raji cells were first incubated at 10^7cells/mL with 10 µM AZDye™ 594-labeled polymer panel for one hour at room temperature. Post-incubation, cells were washed once with PBS. Polymer-treated cells were then incubated with 5 µg/mL helix pomatia lectin-FITC (EY Labs F-3601-1) in 1% BSA in PBS (Mg^{++} and Ca^{++}) on ice for one hour. Cells were then washed twice and

resuspended in 1% BSA in PBS. DAPI (0.1 µg/mL from ThermoFisher # 62247) was added as a live/dead discriminator. Flow cytometry data was acquired with at least 10,000 events for all samples. Polymer-treated cells were prepared in duplicate and unstained, single-color, and polymer-free lectin controls were also prepared. FITC-anti-human CD37 antibody (Biolegends 356304) was used at 10 µg/mL to prepare a FITC single-color control. Data were analyzed by taking median fluorescence intensity (MFI) values associated with lectin binding and polymer coating, normalized against background lectin labeling and efficiency of fluorophore labeling where applicable, normalized against one another to account for differentiating amounts of polymer on cell surface, averaged, and plotted with standard error.

2 Supplementary Figures and Tables

2.1 Supplementary Figures

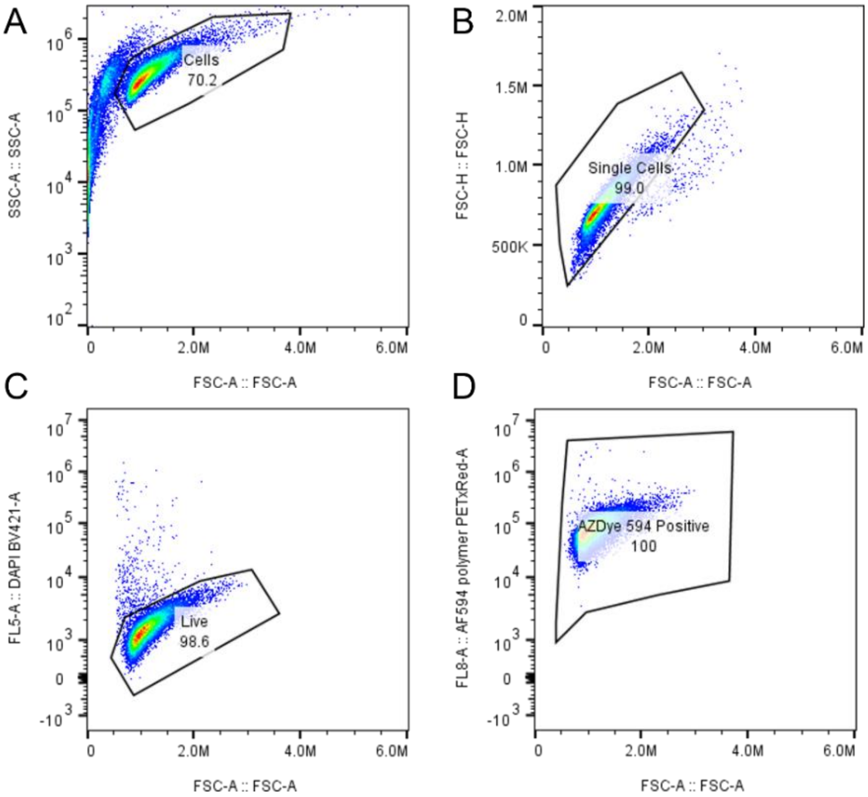

Supplementary Figure 1. General gating tree for flow cytometry data: A) side scatter (SSC) vs. forward scatter (FSC) to exclude cell debris B) FSC-height vs. FSC-area to identify single cells, C) BV421 vs. FSC to exclude dead DAPI-positive cells, and when applicable, D) PE-Texas Red vs. FSC to identify AZ Dye 594-positive cells. Sample data are AZDye^{TM} 594-50% glycosylated 92mer-coated Raji cells, analyzed directly after incubation with polymer.

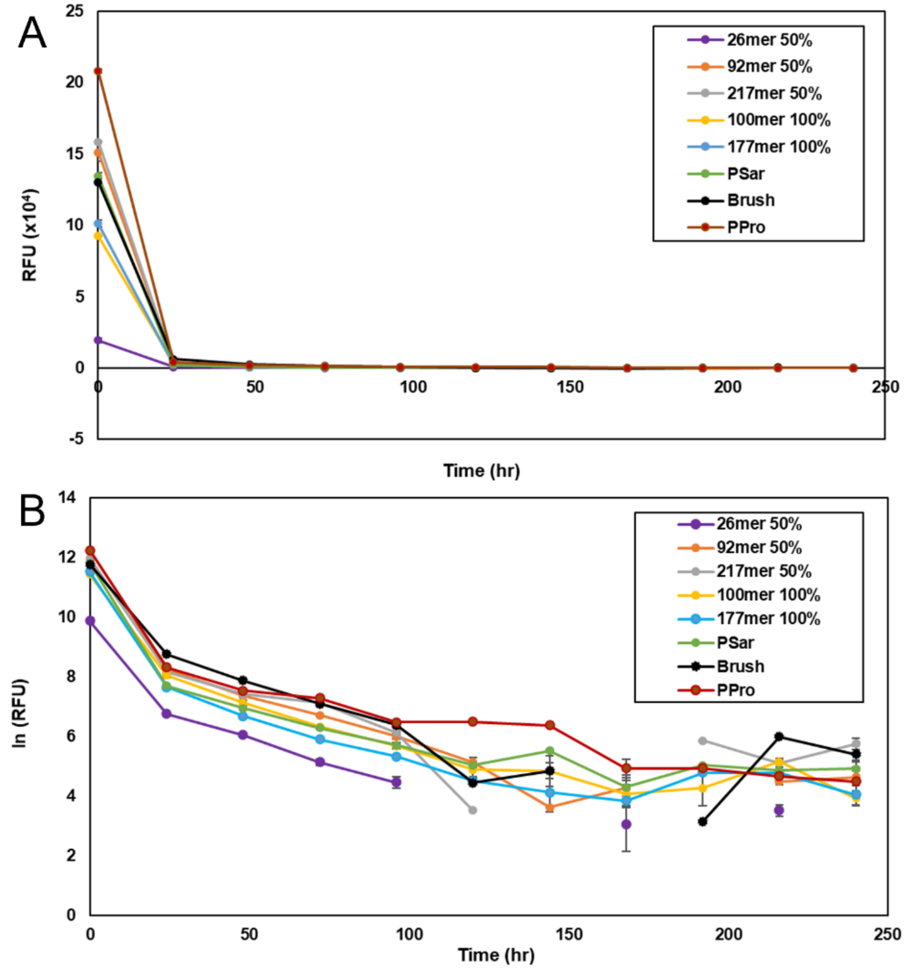

Supplementary Figure 2. A) Relative fluorescence resulting from AZDye^{TM} 594-labeled polymer residence on cell surface vs. time. Same data as in Figure 3B in manuscript, but this plot also includes incorporation density directly post-incubation with polymer (time = 0 hr). B) Natural log plot of the data, demonstrating that polymer residence exponentially decays from ~ days 1-4 while exhibiting faster decay after initial incubation and slower decay after day 4. Data were collected via flow cytometry for 10 days post-incubation with polymer and averaged median fluorescence intensities are plotted with their associated standard error with n = 2.

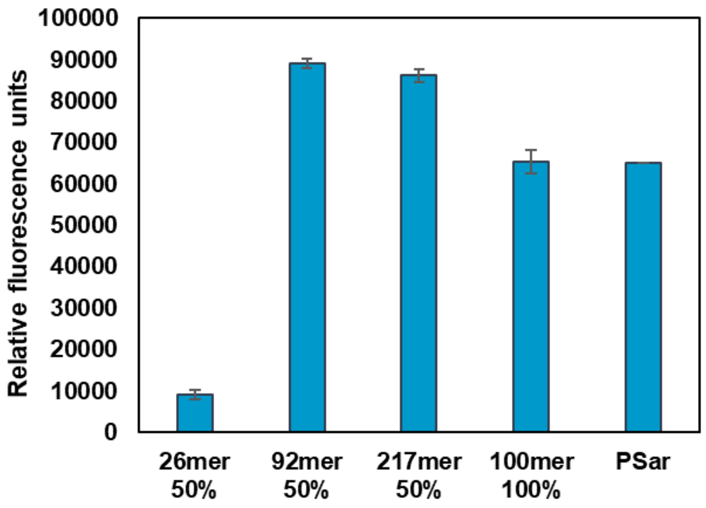

Supplementary Figure 3. Density of incorporation of initial, smaller polymer panel into cell membrane of Raji cells. Raji cells were incubated with AZDye^{TM} 594-labeled polymer for 1 hr and analyzed directly post-incubation via flow cytometry. Averaged median fluorescence intensities are plotted with their associated standard error with n = 2 against polymer type. These data were taken at different voltage settings than those in the manuscript, but densities of incorporation of different polymers trend consistently (Figure 2C). These data are included to indicate reproducibility over multiple experiments.

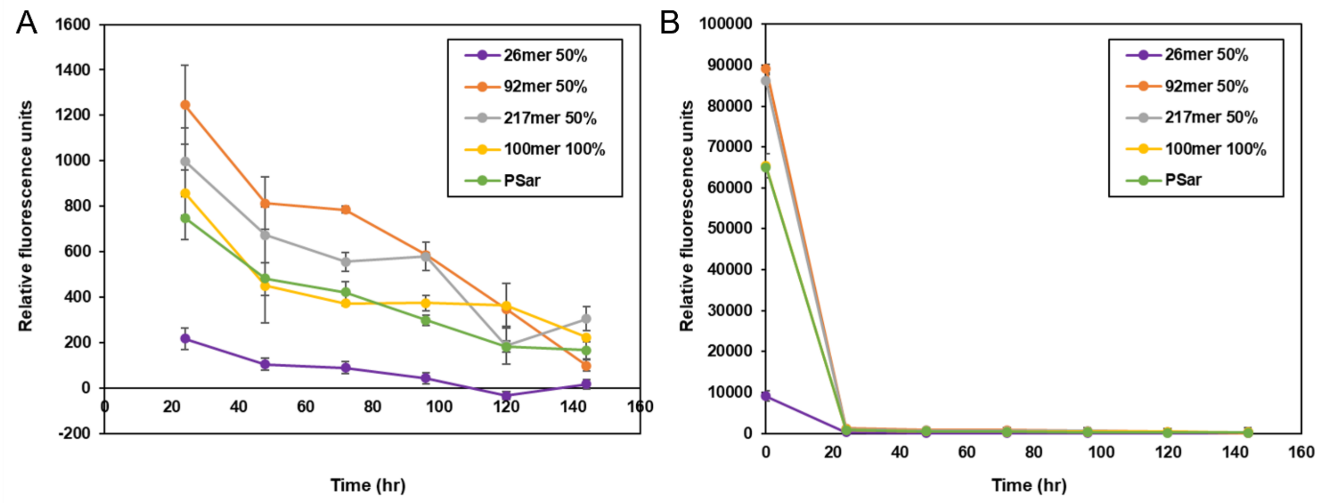

Supplementary Figure 4. Persistence of initial, smaller polymer panel on Raji cells. Plots A) & B) show relative fluorescence resulting from AZDye^{TM} 594-labeled polymer residence on cell surface vs. time B) with or A) without inclusion of data collected directly post incubation (time = 0 hr). Data were collected via flow cytometry for six days post-incubation and averaged median fluorescence intensities are plotted with their associated standard error with n = 2. Continuation of same study as in Figure 3 in SM and data shows similar trends as seen in Figure 3B in manuscript. These data are included to indicate reproducibility over multiple experiments.

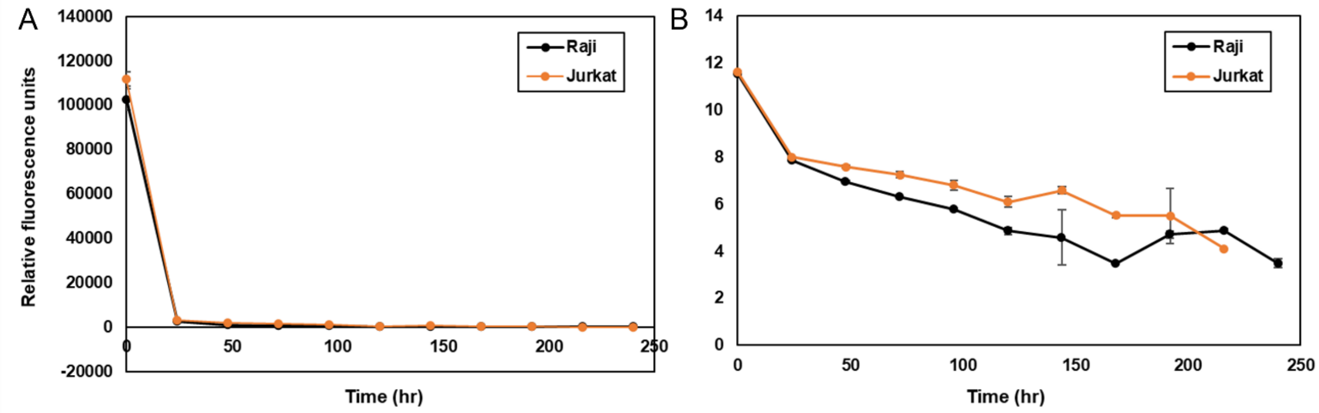

Supplementary Figure 5. A) Plot of relative fluorescence resulting from AZDye^{TM} 594-50% glycosylated 92mer residence on cell surface vs. time. Data shown for two suspension cell types, Raji B lymphocytes and Jurkat T lymphocytes. Same data as in Figure 3D in manuscript, but this plot also includes incorporation density directly post-incubation with polymer (time = 0 hr). B) Natural log plot of the data, demonstrating that polymer residence exponentially decays from ~ days 1-5 while exhibiting faster decay after initial incubation and slower decay after day 5. Data were collected via flow cytometry for 10 days post-incubation with polymer and averaged median fluorescence intensities are plotted with their associated standard error with n = 2.

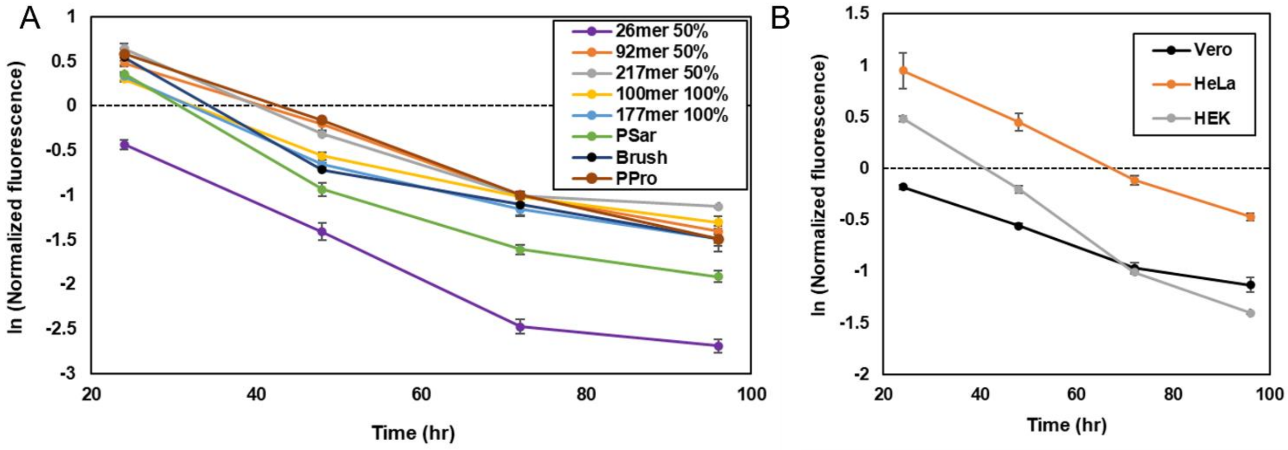

Supplementary Figure 6. Natural log plots of A) residence of full AZDye^{TM}-594-labeled polymer panel on HEK 293 cells vs. time and B) residence of AZDye^{TM} 594-50% glycosylated 92mer on adherent cell panel vs. time. Plots demonstrate that polymer residence exponentially decays from ~ day 1 through day 3 or 4. Adherent cells were incubated with polymer for 1 hr, plated on 24-well plates for adherence, imaged every 24 hrs for four days, and analyzed via thresholding, pixel quantification, and natural log calculation in ImageJ and Excel.

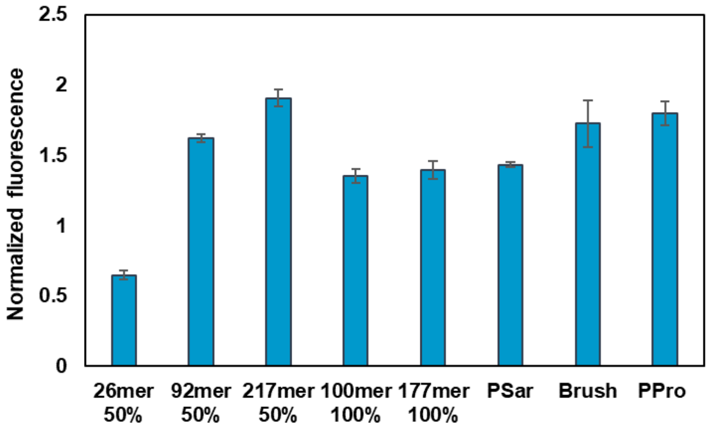

Supplementary Figure 7. Density of incorporation of full polymer panel into cell membrane of HEK 293 cells analyzed via fluorescence imaging 24 hours post-polymer incubation. HEK 293 cells were incubated with AZDye^{TM} 594-labeled polymer for 1 hr, plated on 24-well plates for adherence, imaged one day later, and analyzed via thresholding and pixel quantification in ImageJ. AZDye^{TM} 594 fluorescence was normalized to number of cells (via cell margin quantification) and plotted against time. All trends consistent with flow cytometry data collected directly post-incubation with polymer, with AZDye^{TM} 594-100% glycosylated 177mer being a potential outlier. These data are included to indicate reproducibility over multiple experiments.

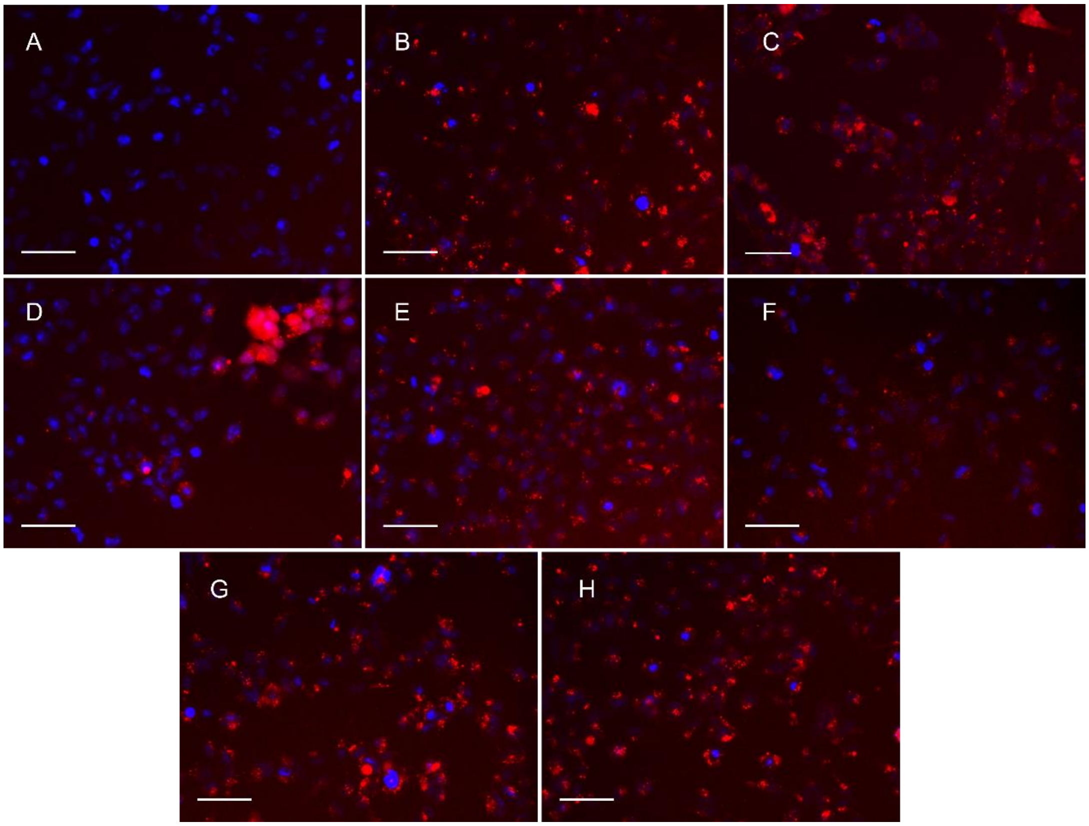

Supplementary Figure 8. Fluorescent images, taken at 20x magnification, of HEK 293 cells coated with A) no polymer or AZDye^{TM} 594-labeled B) polySar$_{100}$, C) polyPro$_{90}$ D) 50% glycosylated 26mer E) 50% glycosylated 92mer, F) 50% glycosylated 217mer, G) 100% glycosylated 100mer, and H) 100% glycosylated 177mer. Cells were nuclear stained with Hoescht 33342 and imaged 24 hours post incubation with polymer. Scale bars are 50 µm.

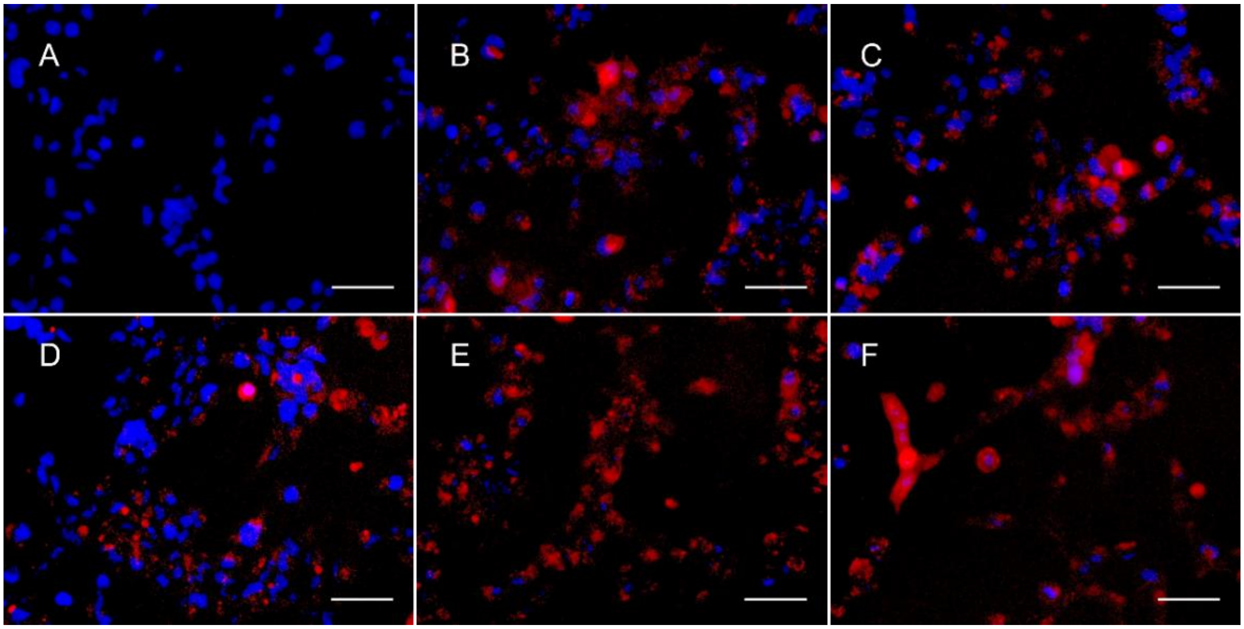

Supplementary Figure 9. Fluorescent images, taken at 20x magnification, of HEK 293 cells coated with initial, smaller polymer panel: A) no polymer and AZDyeTM 594-labeled B) 50% glycosylated 26mer , C) 50% glycosylated 92mer, D) 50% glycosylated 217mer, E) 100% glycosylated 100mer, or G) polySar$_{100}$. Cells were nuclear stained with Hoescht 33342 and imaged 24 hours post incubation with polymer. Scale bars are 50 µm. These data are included to indicate reproducibility over multiple experiments.

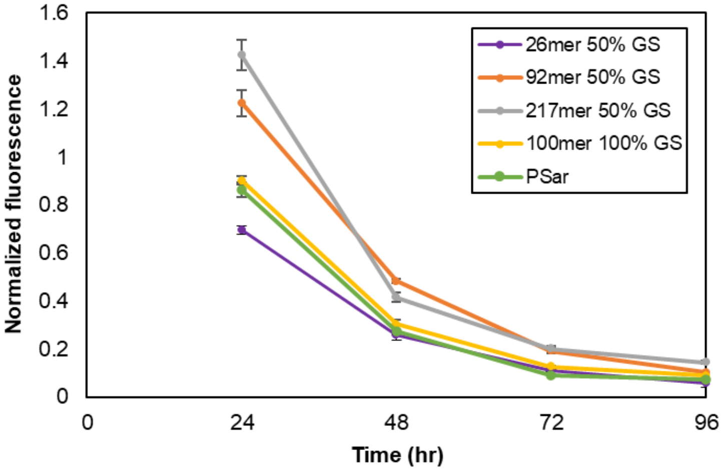

Supplementary Figure 10. Persistence of initial, smaller polymer panel on HEK 293 cells. HEK 293 cells were incubated with AZDye^{TM} 594-labeled polymer for 1 hr, plated on 24-well plates for adherence, imaged every 24 hr for 3 days post-polymer incubation, and analyzed via thresholding and pixel quantification in ImageJ. AZDye^{TM} 594 fluorescence was normalized to number of cells (via cell margin quantification) and plotted against time. Trends similar to persistence studies with full polymer panel in manuscript (Figure 3A). These data are included to indicate reproducibility over multiple experiments.

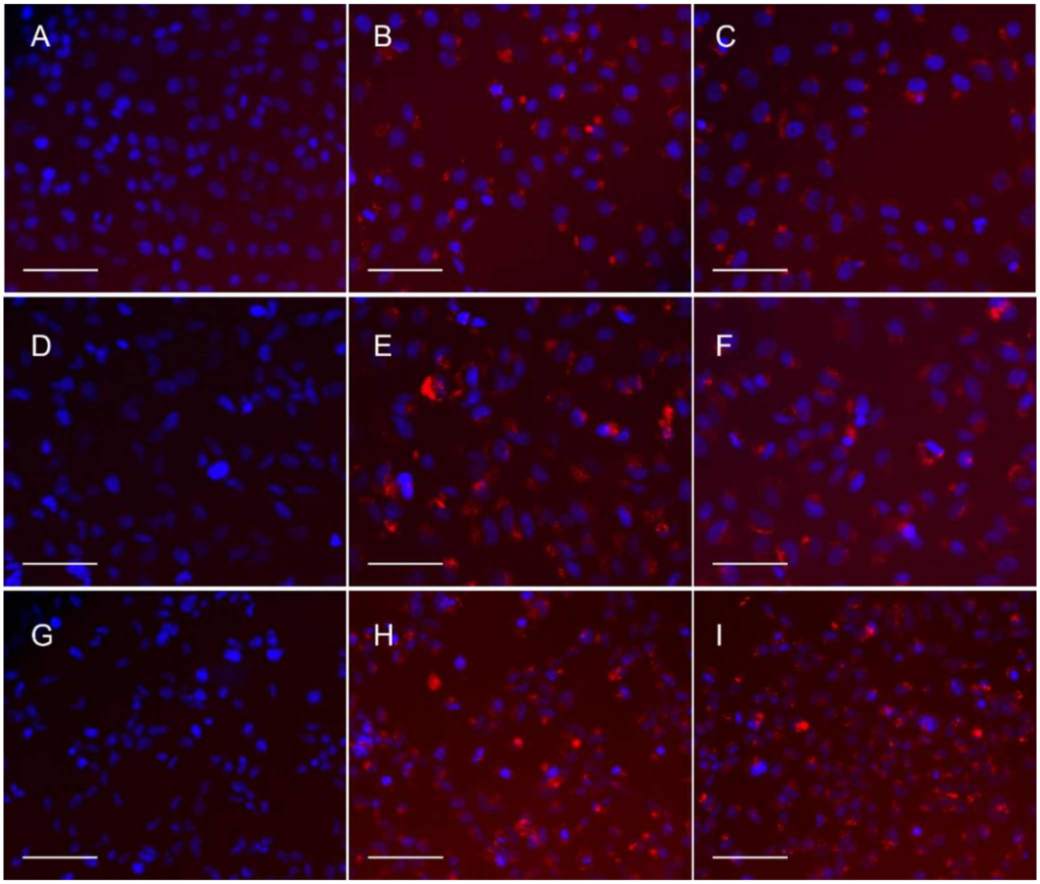

Supplementary Figure 11. Fluorescent images, taken at 20x magnification, of different adherent cells coated with 10 µM AZDye™ 594-50% glycosylated 92mer (B, C, E, F, H, I) as compared to uncoated cells (A, D, G). Cells were nuclear stained with Hoescht 33342 and imaged 24 hours post incubation with glycopolymer. Cell types are as follows: A-C) Vero, D-F) HeLa, and G-I) HEK 293. Scale bars are 75 µm.

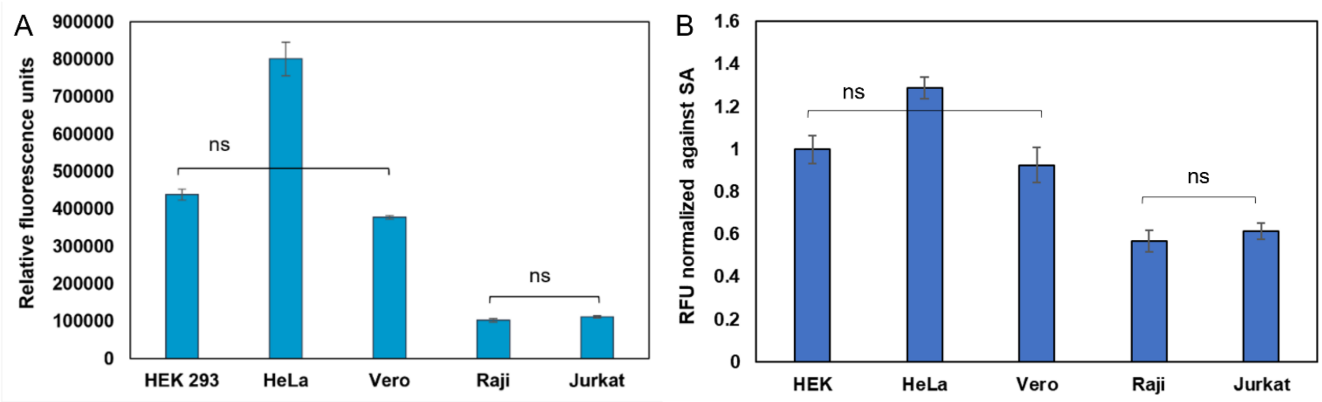

Supplementary Figure 12. A-B) Density of incorporation of AZDye™ 594-50% glycosylated 92mer into cell membranes of both adherent and suspension cell types. Data were collected via flow cytometry directly post-incubation with polymer and averaged median fluorescence intensities are plotted with their associated standard error with n = 2 against cell type. A) Data corresponds to that in Figure 2D of manuscript but with no cell-size normalization. B) Data corresponds to that in Figure 2D of manuscript but is normalized against surface area.

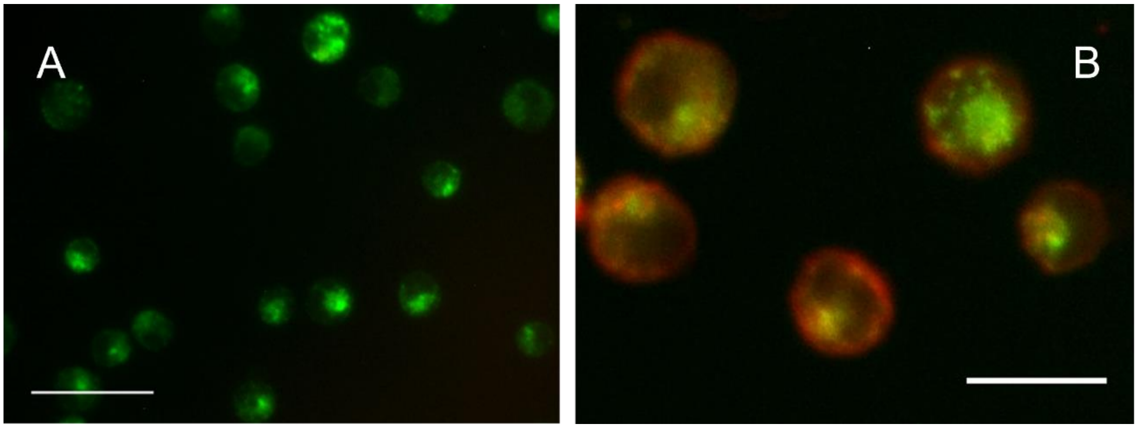

Supplementary Figure 13. A) CF488A-transferrin labeled HEK 293 control with no polymer coating. CF488A-transferrin was incubated at 30 µg/mL with HEK 293 cells at room temperature for 30 minutes, followed by a 15-minute 37°C incubation step to allow transferrin to be uptaken. Cells were washed and fixed. Images taken at 40x. Scale bar is 50 µm. B) Close-up of polymer-coated, transferrin-labeled HEK 293 cells from Fig. 4C in manuscript. HEK cells were incubated with 10 µM AZDye^{TM} 594-50% glycosylated 92mer for one hour and CF488A-transferrin was added to a final concentration of 30 µg/mL for the last 30 minutes of the incubation. Cells were then incubated at 37°C for transferrin uptake, washed, and fixed. Scale bar is 25 µm.

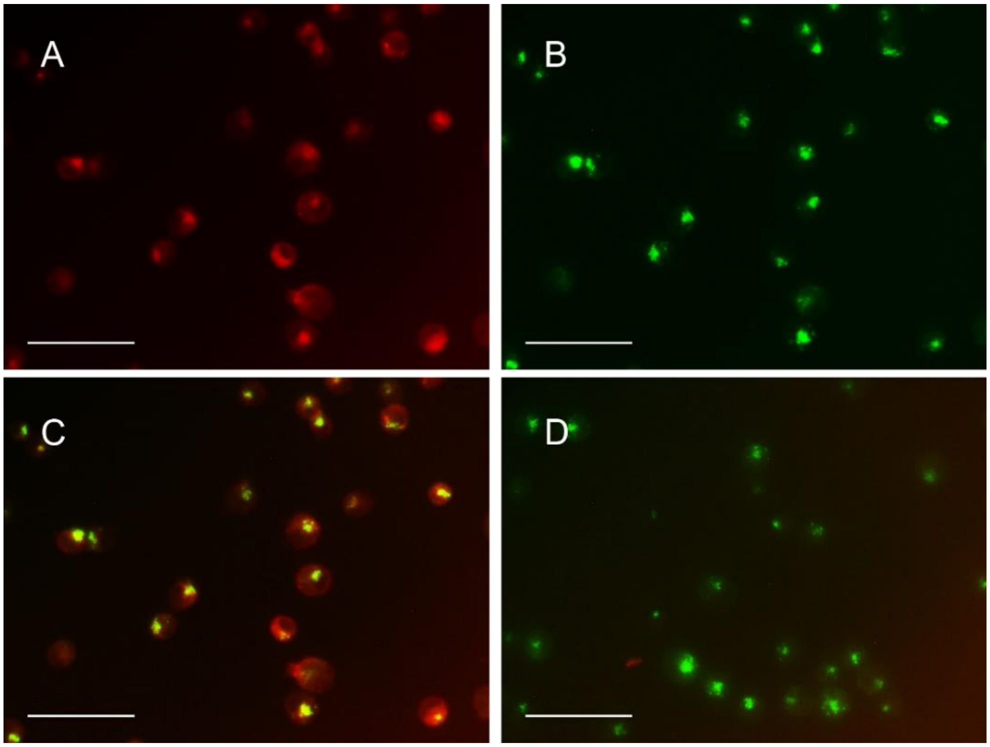

Supplementary Figure 14. Fluorescent images taken at 40x of CF488A-transferrin labeled Raji cells coated with D) no polymer or A-C) AZDye^{TM} 594-50% glycosylated 92mer, where A) is glycopolymer, B) is transferring, and C) is the overlay. Raji cells were incubated with 10 µM AZDye^{TM} 594-50% glycosylated 92mer for one hour and CF488A-transferrin was added to a final concentration of 30 µg/mL for the last 30 minutes of the incubation. Cells were then incubated at 37°C for transferrin uptake, washed, and fixed. Images taken at 40x. Scale bars are 50 µm.

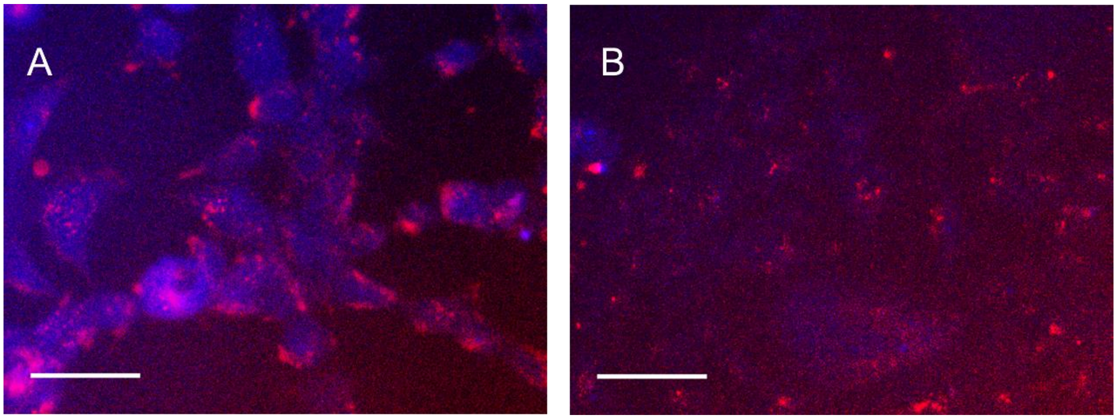

Supplementary Figure 15. Fluorescent images taken at A) 24 hours and B) 48 hours after simultaneous labeling and coating of HEK 293 labeled with CellTracker Blue and 10 µM AZDye™ 594-50% glycosylated 92mer respectively. Polymer is visually well-distributed among cells and its density on the cell surface decreases as CellTracker dye dims. Images taken at 20x magnification. Scale bars are 50 µm.

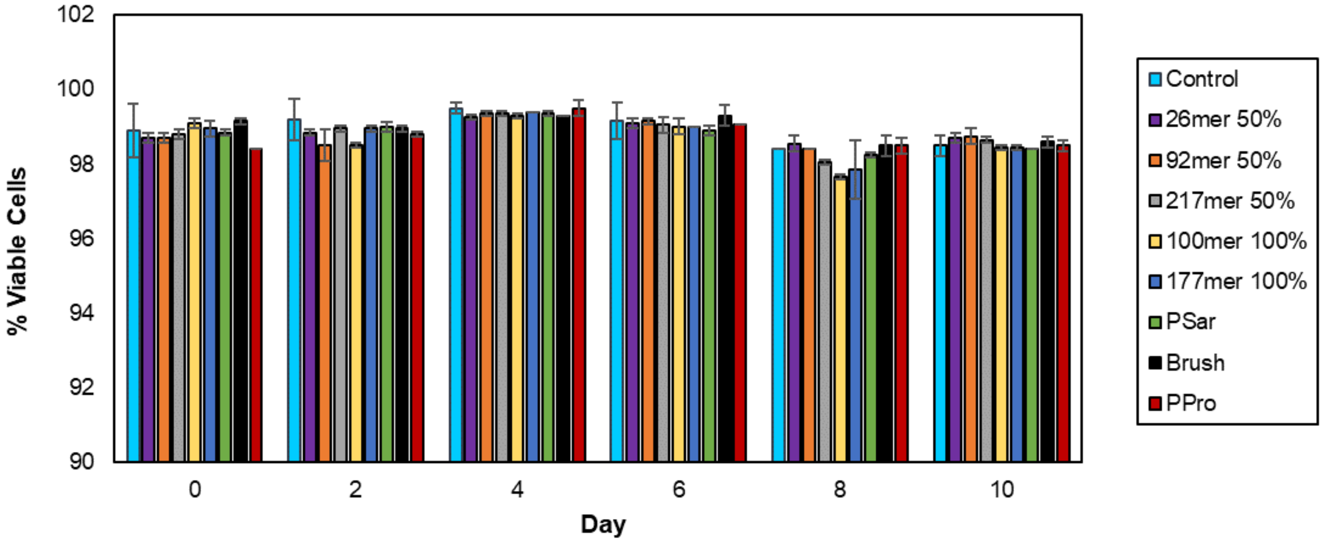

Supplementary Figure 16. Comparison of cell viability as affected by time and the polymer present on the cell surface. Raji cells were labeled Day 0 with 10 µM polymer panel and evaluated every other day for a period of 10 days via flow cytometry. Cells were stained with DAPI as a live/dead discriminator to determine cell viability (dead cells = DAPI +). No evident trends correlating polymer type with decrease in viability.

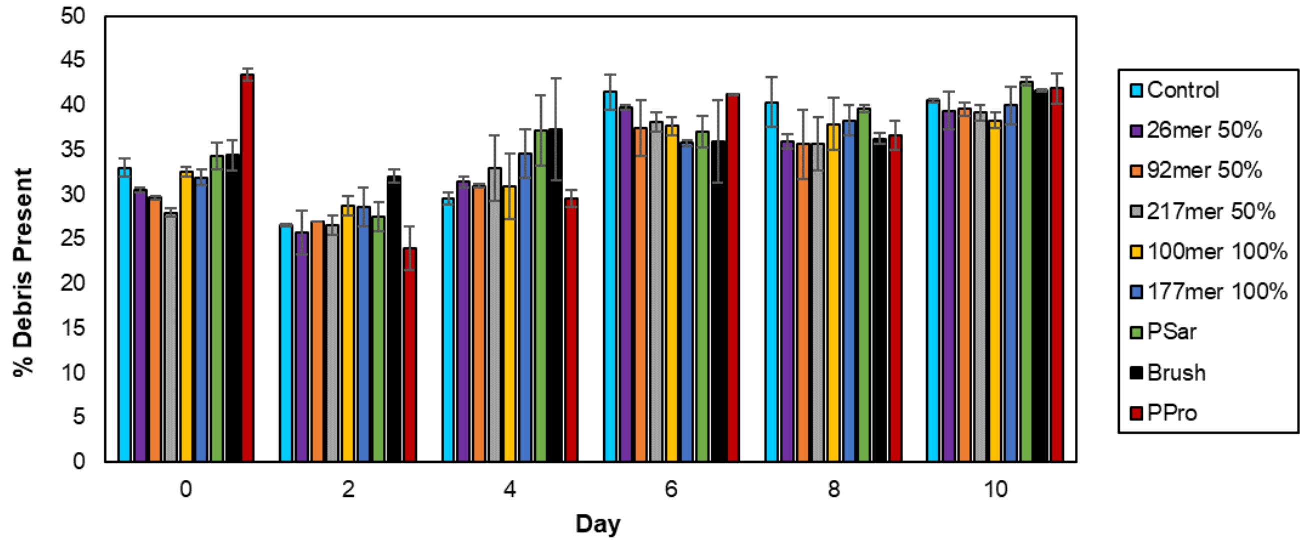

Supplementary Figure 17. Comparison of the amount of cell debris present as affected by time and the polymer present on the cell surface. Raji cells were labeled Day 0 with 10 µM polymer panel and evaluated every other day for a period of 10 days via flow cytometry and associated gating. No evident trends correlating polymer type with debris increase. Debris, as expected, increases over time in all cells, independent of residing polymer.

A

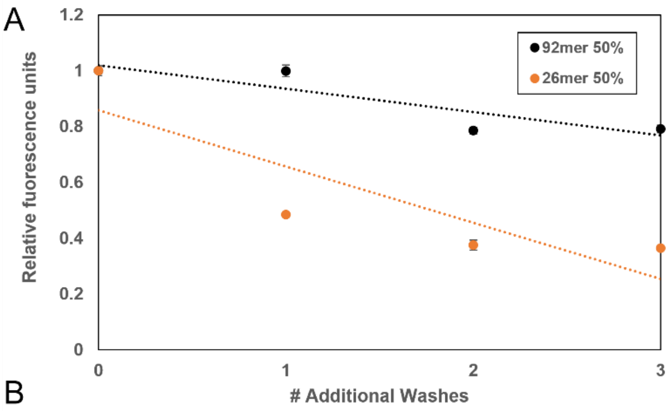

B

Percent of Polymer Present on Cell Surface After Additional PBS Washes				
PBS Wash Number	0	1	2	3
92mer 50%	100 ± 1.2	99.9 ± 2.1	78.6 ± 1.2	79.1 ± 1.4
26mer 50%	100 ± 1.8	48.4 ± 0.4	37.5 ± 1.8	36.5 ± 0

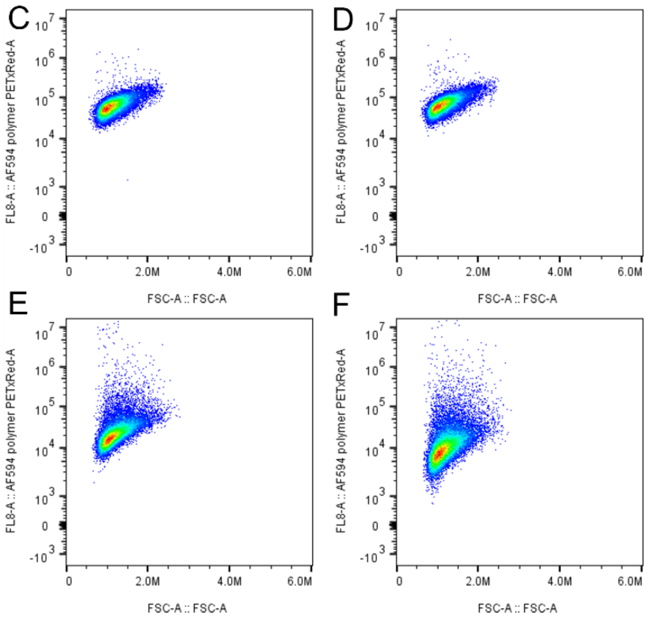

Supplementary Figure 18. Data showing effect of additional PBS washes on polymer density on cell surface. A) compares density of incorporation vs. additional washing of our lowest-density polymer, AZDye™ 594-50% glycosylated 26 mer with AZDye™ 594-50% glycosylated 92mer, which has comparable density to all other polymers. B) outlines the amount retained on the cell surfaces post-washing. It is evident that 50% glycosylated 26mer is particularly susceptible to washing (almost 64% loss vs. 21% loss of 50% glycosylated 92mer. C-F) show comparison of cell populations labeled either with C-D) 50% glycosylated 92mer or E-F) 50% glycosylated 26mer after C) & E) initial wash or D) & F) first additional wash. Cells coated with 50% glycosylated 92mer have little spread increase after wash, while 50% glycosylated 26mer has inherently more spread and also is enhanced with washing.

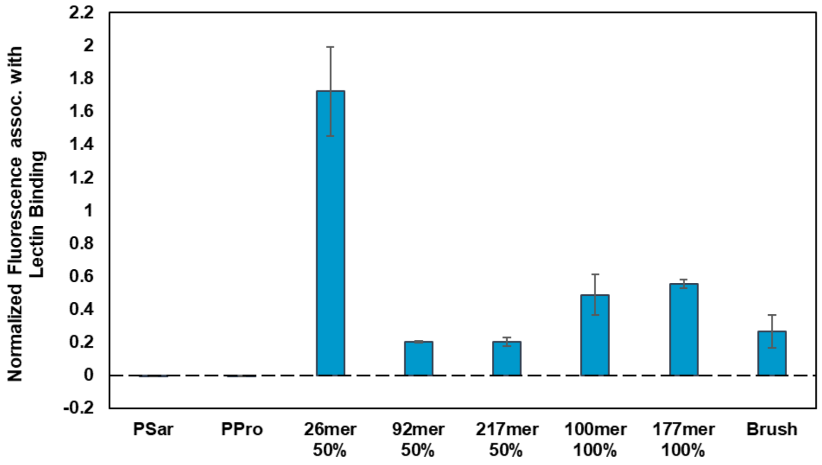

Supplementary Figure 19. Helix pomatia agglutinin binding efficiency to polymers in panel, collected via flow cytometry. Plot shows median fluorescence intensity (MFI) values associated with lectin binding that were normalized against background lectin labeling and against efficiency of polymer coating and averaged. Associated standard error is also plotted. Data corresponds to that in Figure 5A in manuscript, but this plot also encompasses data for the 50% glycosylated 26mer, which is less densely populated on cell surface.

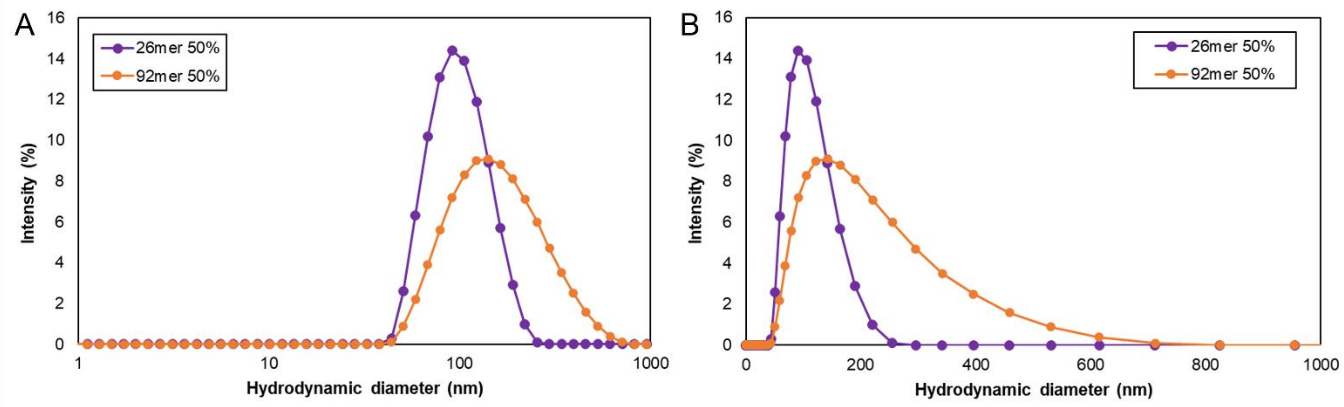

Supplementary Figure 20. Dynamic light scattering curves for 50% glycosylated 26mer and 92mer, shown with A) linear X-axis or B) logarithmic X-axis. Polymers were suspended in 1X DPBS at 1µM and analyzed on a a Malvern Zetasizer Nano ZS (backscatter at angle of 173°).

2.2 Supplementary Tables

Polypeptide-CholA	Molecular Weight (kDa)	αGalNAcSer residues per chain	Fluorophore Labeling efficiency (AZ594)
poly(αGalNAcSer$_{0.5}$-*s*-Ala$_{0.25}$-*s*-Glu$_{0.25}$)$_{26}$ 50% glycosylated 26mer	5.9	13	100 %
poly(αGalNAcSer$_{0.5}$-*s*-Ala$_{0.25}$-*s*-Glu$_{0.25}$)$_{92}$ 50% glycosylated 92mer	18	46	52 %
poly(αGalNAcSer$_{0.5}$-*s*-Ala$_{0.25}$-*s*-Glu$_{0.25}$)$_{217}$ 50% glycosylated 92mer	43.2	108	28 %
poly(αGalNAcSer)$_{100}$ 100% glycosylated 100mer	30	100	81.4 %
poly(αGalNAcSer)$_{177}$ 100% glycosylated 177mer	52	177	94.7 %
(polyGlu$_{0.8}$-*s*-polyAIK$_{0.2}$)$_{50}$-*g*-αGalNAcSer$_{13}$ glycobrush	44	130	34.1 %
polySar$_{100}$	8	0	100 %
polyPro$_{90}$	9	0	85.9%

Table 1. Structures used in this study, their molecular weights, and the number of glycans per chain. The efficiency of AZDyeTM 594 labeling of the polymer panel used in this study. All flow cytometry and imaging data collected was normalized against labeling efficiency.

Polymer	Cell Type	Half-life
26mer 50%	Raji	36.0 ± 1.8
92mer 50%	Raji	38.0 ± 0.3
217mer 50%	Raji	61.5 ± 1.0
100mer 100%	Raji	59.8 ± 13.5
PSar$_{100}$	Raji	53.7 ± 0.2
26mer 50%	HEK	20.4 ± 0.4
92mer 50%	HEK	22.7 ± 2.1
217mer 50%	HEK	24.7 ± 2.1
100mer 100%	HEK	26.6 ± 3.5
PSar$_{100}$	HEK	20.8 ± 1.1

Table 2. Half-life and associated standard error for all polymers that were run in our initial panel as well as our final, published panel.

	Raji Cells			HEK 293 Cells		
**Treatments pair**	**Q statistic**	**p-value**	**Tukey HSD inference**	**Q statistic**	**p-value**	**Tukey HSD inference**
26mer 50% vs. 92mer 50%	30.3862	0.001005	** p<0.01	25.8894	0.0010053	** p<0.01
26mer 50% vs. 217mer 50%	32.1104	0.001005	** p<0.01	28.6996	0.0010053	** p<0.01
26mer 50% vs. 100mer 100%	16.8455	0.001005	** p<0.01	16.1225	0.0010053	** p<0.01
26mer 50% vs. 177mer 100%	18.8455	0.001005	** p<0.01	26.4120	0.0010053	** p<0.01
26mer 50% vs. PSar	26.5417	0.001005	** p<0.01	12.3187	0.0010053	** p<0.01
26mer 50% vs. Brush	25.5475	0.001005	** p<0.01	31.8011	0.0010053	** p<0.01
26mer 50% vs. PPro	43.5031	0.001005	** p<0.01	22.4997	0.0010053	** p<0.01
92mer 50% vs. 217mer 50%	1.7242	0.899995	insignificant	2.8103	0.5372298	insignificant
92mer 50% vs. 100mer 100%	13.5407	0.001005	** p<0.01	9.7669	0.0017914	** p<0.01
92mer 50% vs. 177mer 100%	11.5407	0.001005	** p<0.01	0.5227	0.8999947	insignificant
92mer 50% vs. PSar	3.8445	0.240228	insignificant	13.5707	0.0010053	** p<0.01
92mer 50% vs. Brush	4.8387	0.099155	insignificant	5.9118	0.0377672	insignificant
92mer 50% vs. PPro	13.1169	0.001005	** p<0.01	3.3897	0.3513970	insignificant
217mer 50% vs. 100mer 100%	15.2649	0.001005	** p<0.01	12.5771	0.0010053	** p<0.01
217mer 50% vs. 177mer 100%	13.2649	0.001005	** p<0.01	2.2876	0.7165492	insignificant
217mer 50% vs. PSar	5.5687	0.051283	insignificant	16.3809	0.0010053	** p<0.01
217mer 50% vs. Brush	6.5629	0.021401	insignificant	3.1015	0.4404328	insignificant
217mer 50% vs. PPro	11.3926	0.001005	** p<0.01	6.1999	0.0293121	insignificant
100mer 100% vs. 177mer 100%	2	0.815221	insignificant	10.2896	0.0012554	** p<0.01
100mer 100% vs. PSar	9.6962	0.001884	** p<0.01	3.8038	0.2487985	insignificant
100mer 100% vs. Brush	8.702	0.00385	** p<0.01	15.6786	0.0010053	** p<0.01
100mer 100% vs. PPro	26.6576	0.001005	** p<0.01	6.3772	0.0251251	* p<0.05
177mer 100% vs. PSar	7.6963	0.008366	** p<0.01	14.0933	0.0010053	** p<0.01
177mer 100% vs. Brush	6.702	0.019001	insignificant	5.3891	0.0602801	insignificant
177mer 100% vs. PPro	24.6576	0.001005	** p<0.01	3.9123	0.2266877	insignificant
PSar vs. Brush	0.9942	0.899995	insignificant	19.4824	0.0010053	** p<0.01
PSar vs. PPro	16.9613	0.001005	** p<0.01	10.1810	0.0013485	** p<0.01
Brush vs. PPro	17.9556	0.001005	** p<0.01	9.3014	0.0024851	** p<0.01

Table 3. Tukey test statistics for density studies with HEK and Raji cells. Significance at $p < 0.01$.

2.3 NMR Spectra

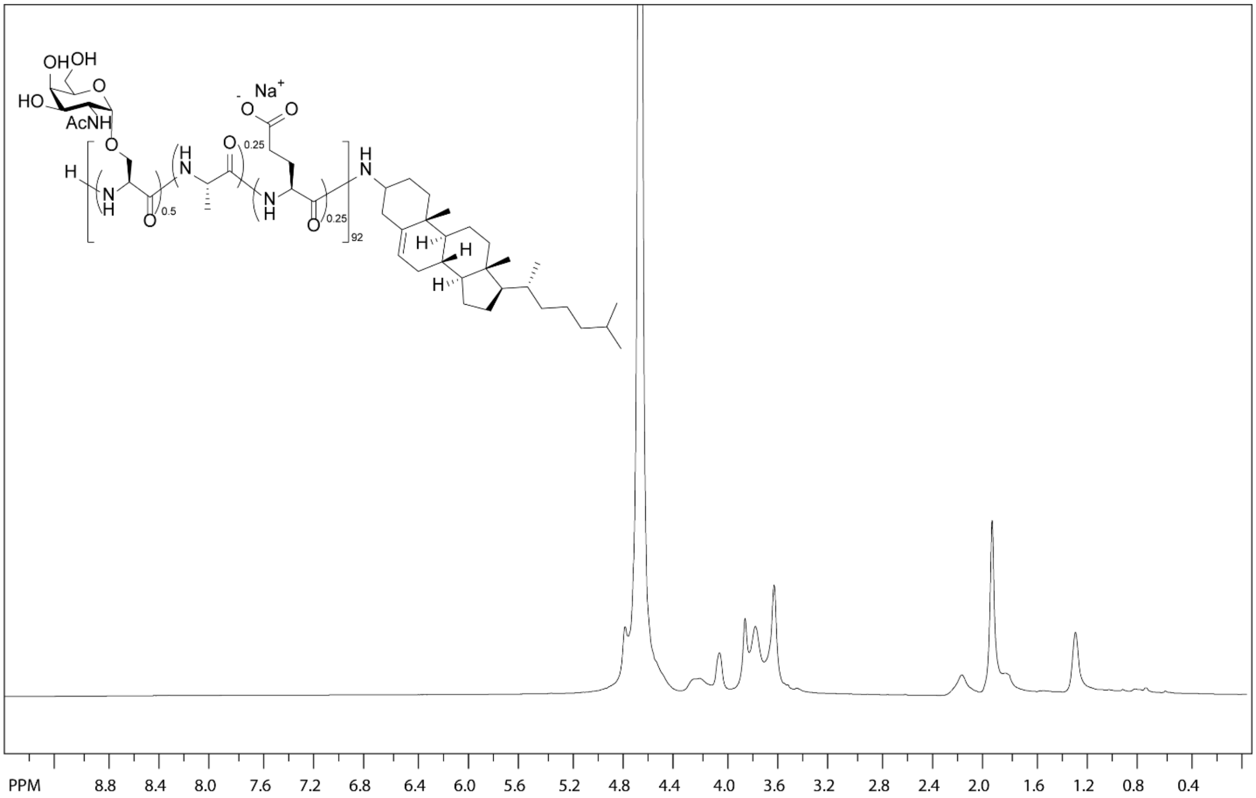

3 References

(1) Detwiler, R. E.; Schlirf, A. E.; Kramer, J. R. Rethinking Transition Metal Catalyzed N-Carboxyanhydride Polymerization: Polymerization of Pro and AcOPro N-Carboxyanhydrides. *J. Am. Chem. Soc.* **2021**, *143* (30), 11482–11489.

(2) Clauss, Z. S.; Kramer, J. R. Polypeptoids and Peptoid-Peptide Hybrids by Transition Metal Catalysis. *ACS Appl. Mater. Interfaces* **2021**.

(3) Kramer, J. R.; Onoa, B.; Bustamante, C.; Bertozzi, C. R. Chemically Tunable Mucin Chimeras Assembled on Living Cells. *Proc. Natl. Acad. Sci.* **2015**, *112* (41), 12574–12579.

(4) Clauss, Z. S.; Wardzala, C. L.; Schlirf, A. E.; Wright, N. S.; Saini, S. S.; Onoa, B.; Bustamante, C.; Kramer, J. R. Tunable, Biodegradable Grafting-from Glycopolypeptide Bottlebrush Polymers. *Nat. Commun.* **2021**, *12* (1), 6472.
